# Molecular dosimetry of estragole and 1′-hydroxyestragole-induced DNA adduct formation, clastogenicity and cytotoxicity in human liver cell models

**DOI:** 10.1007/s00204-025-04084-2

**Published:** 2025-05-21

**Authors:** G. Ackermann, M. Peil, C. Quarz, A. Schmidt, M. Halaczkiewicz, A. D. Thomas, S. Stegmüller, E. Richling, G. Manolikakes, M. Christmann, J. H. Küpper, D. Schrenk, J. Fahrer

**Affiliations:** 1 Division of Food Chemistry and Toxicology, Department of Chemistry, RPTU Kaiserslautern-Landau, Erwin-Schrödinger-Str. 52, 67663 Kaiserslautern, Germany; 2 Institute of Toxicology, University Medical Center, Obere Zahlbacher Str. 67, 55131 Mainz, Germany; 3 Division of Organic Chemistry, Department of Chemistry, RPTU Kaiserslautern-Landau, Erwin-Schrödinger-Str. 54, Kaiserslautern, Germany; 4 School of Applied Sciences, University of the West of England, Bristol, BS16 1QY UK; 5 Division of Molecular Cell Biology, Faculty of Environment and Nature Science, Brandenburg University of Technology Cottbus-Senftenberg, Universitätsplatz 1, 01968 Senftenberg, Germany

**Keywords:** Plant toxin, Phenylpropanoids, Estragole, Human hepatocytes, Liver damage, Cytotoxicity, Genotoxicity, Clastogenicity, Concentration–response modelling

## Abstract

The phenylpropene estragole (ES) is found in essential oils of herbs and spices, such as bitter fennel and basil. Humans are exposed to ES through the diet and phytomedicines. After its absorption, ES undergoes metabolic activation by CYP1A2 and SULT1A1 in the liver, which can give rise to DNA adducts and hepatocarcinogenesis. Until now, quantitative genotoxicity data for ES in human liver cells are scarce, correlating DNA adduct levels with critical effects such as clastogenicity. Here, we used human HepG2 and HepG2-CYP1A2 cells as well as primary human hepatocytes (PHH) to study the genotoxic, clastogenic and cytotoxic potential of ES and its crucial metabolite 1′-hydroxyestragole (1′OH-ES). In addition, primary rat hepatocytes (PRH) were used for selected endpoints. Treatment of HepG2-CYP1A2 cells with ES (0–2 mM) led to the concentration-dependent formation of E3′-*N*^2^-dG adducts. Apart from a moderate γH2AX induction, neither p53 accumulation nor cytotoxicity was observed. However, clastogenicity was demonstrated at ES concentrations ≥ 1 mM. Incubation of HepG2 cells with 1′OH-ES (0—35 µM) led to 10–50-fold higher E3′-*N*^2^-dG adduct levels compared to equimolar ES concentrations. Furthermore, 1′OH-ES caused γH2AX formation, p53 accumulation and cytotoxicity, which was confirmed in PHH. In agreement, 1′OH-ES induced clastogenicity at concentrations ≥ 25 µM. Molecular dosimetry revealed that a certain E3′-*N*^2^-dG adduct level is required to trigger clastogenicity and cytotoxicity. This was confirmed by Benchmark Concentration (BMC) modelling, showing that the BMC for clastogenicity is 12–17-fold higher than the respective BMC for DNA adduct formation. Our data indicate that a threshold level of DNA adducts is required, both in rat and human liver cells, to trigger markers of clastogenicity. These levels are unlikely to be reached in humans following chronic ES exposure through phytomedicines or the diet.

**Supplementary Information:**

The online version contains supplementary material available at 10.1007/s00204-025-04084-2.

## Introduction

Estragole (ES) belongs to the group of phenylpropenes, which are secondary plant constituents found in various herbs and spices (Atkinson 2018). ES occurs mainly in the essential oil of bitter fennel (3.5–12%), sweet basil (20–89%) and tarragon (60–75%) (EMA/HMPC 2022). Therefore, the human diet and herbal medicinal products, e.g., bitter fennel tea infusions, are relevant sources for exposure. ES content is known to depend on the raw product, the processing of the fennel fruits as well as the preparation method for tea infusions. The reported extraction efficiencies of ES from fennel range from below 0.1 up to 12%, resulting in ES concentrations of 0.05–4.64 mg/L (0.34–31.3 µM) in fennel tea infusions (van den Berg et al. 2014; Zeller and Rychlik 2006). The average daily ES intake was estimated to range between 0.5 and 5 mg per person (EMA/HMPC 2022; Punt et al. 2016).

After oral uptake, the lipophilic ES is rapidly absorbed and transported to the liver (Anthony et al. 1987). ES then undergoes metabolic activation by hydroxylation at the 1′-position of the allylic side chain to form the proximal carcinogen 1′OH-ES (Rietjens et al. 2014), which has recently been modelled in silico with regard to the reaction energy profiles (Yadav et al. 2021) This critical step is catalysed predominantly by CYP1A2 and also CYP2A6 (Jeurissen et al. 2007). The formed metabolite is further conjugated with sulphate mediated by SULT1A1 and SULT1C2, which is similar to the metabolic activation of methyleugenol (ME) and safrole (Herrmann et al. 2012; Honda et al. 2016; Suzuki et al. 2012b). The formed 1′-sulfooxy-metabolite spontaneously decomposes, which gives rise to a reactive carbenium ion. This attacks nucleophilic centres in DNA, causing E3′-*N*^2^-dG and E3′-*N*^6^-dA as main adducts (Ishii et al. 2011; Wiseman et al. 1987). In contrast to this metabolic activation pathway, which occurs preferentially at high ES concentrations, ES as well as its 1′-OH-metabolite can be detoxified by *O*-demethylation as main detoxification pathway in rodents (Anthony et al. 1987). The demethylated metabolites are further glucuronidated or conjugated with glutathione, promoting subsequent urinary excretion (Drinkwater et al. 1976; Iyer et al. 2003). Another metabolic pathway is via the epoxidation of the allylic side chain (Miller et al. 1983; Wiseman et al. 1987), producing a potentially genotoxic epoxide in vitro. However, in vivo this phase I-metabolite was shown to be detoxified by epoxide hydrolases and via conjugation with glutathione (Guenthner and Luo 2001; Luo et al. 1992).

Similar to ME and safrole, ES was shown to cause liver tumours in rodents (Drinkwater et al. 1976; Miller et al. 1983). While ME and safrole were classified by the International Agency for Research on Cancer (IARC) as “probably carcinogenic to humans” (IARC 2A) and “possibly carcinogenic to humans” (IARC 2B) (IARC 1987, 2024), respectively, ES has not been classified by IARC so far. However, the Scientific Committee on Food (SCF) evaluated ES as genotoxic and carcinogenic, with the recommendation to reduce the exposure of humans to ES and to restrict its use (Scientific Committee on Food 2001). As a consequence, the flavouring of food with ES, ME, and safrole in their isolated form had been prohibited since 2011 in the EU (EC 2008). In their recent public statement, the Committee on Herbal Medicinal Products (HMPC) of the European Medicines Agency (EMA) concluded that ES is a genotoxic carcinogen in rodents (EMA/HMPC 2022).

DNA adduct formation in the liver is considered an initiating event for ES-triggered carcinogenicity. A recent study in HepaRG cells provided evidence that E3′-*N*^2^-dG adducts are only partially repaired and persist, which can result in DNA adduct accumulation after repeated exposure (Yang et al. 2020, 2021). Another study performed in primary rat hepatocytes (PRH) revealed maximum E3′-*N*^2^-dG adduct formation after 6 h followed by a decrease over 48 h, which was also observed for the minor E3′-*N*^6^-dA adduct (Schulte-Hubbert et al. 2020). Subsequent modelling of the concentration–response data indicated a practical threshold for DNA adduct formation at 0.5 µM ES (Schulte-Hubbert et al. 2020). This raises the question whether a point of departure (PoD) also exists for the clastogenicity and cytotoxicity triggered by ES or its critical phase I-metabolite 1′OH-ES in human liver cells. The need for more quantitative genotoxicity data was highlighted in the recent public HMPC statement (EMA/HMPC 2022).

To this end, we used different human liver cell models, including parental HepG2 cells, HepG2 cells stably transduced with CYP1A2 and primary human hepatocytes (PHH), to assess the genotoxic, clastogenic and cytotoxic potential of ES and 1′OH-ES in a concentration-dependent manner. In addition, primary rat hepatocytes (PRH) were used for selected endpoints. First, we measured the levels of the two main DNA adducts E3′-*N*^2^-dG and E3′-*N*^6^-dA as well as DNA strand break induction using the alkaline comet assay. Subsequently, DNA damage response markers (γH2AX, p53) were determined by western blot analysis, whilst cytotoxicity was assessed by the resazurin reduction assay. Furthermore, clastogenicity was studied using the micronucleus assay. Finally, the concentration–response data were subject to benchmark concentration modelling.

## Materials and methods

### Cell culture

HepG2 cells (#ACC 180, DSMZ, Braunschweig, Germany) and HepG2 cells stably transduced with human CYP1A2 (clone 7) were used (Steinbrecht et al. 2020). Notably, the HepG2-CYP1A2 cell line was demonstrated to exhibit stable CYP1A2 protein expression and high enzyme activity as revealed by the conversion of phenacetin to paracetamol (Steinbrecht et al. 2020). Both cell lines were cultivated in DMEM high glucose without pyruvate (Gibco, Thermo Fisher Scientific, Waltham MA, USA) supplemented with 10% foetal calf serum (FCS, Pan-Biotech, Aidenbach, Germany) and 1% penicillin/streptomycin (Gibco, Thermo Fisher Scientific, Waltham MA, USA). Cell lines were mycoplasma negative, as demonstrated by routine PCR testing using Venor®GeM OneStep (Berlin, Germany). Cryopreserved PHH (BioIVT, Brussels, Belgium) pooled from 10 donors (5 × females, 5 × males) were handled according to the protocol of the manufacturer. In brief, cells were thawed, directly suspended in INVITROGRO™ CP Medium (BioIVT, Brussels, Belgium), and counted using trypan blue to determine the cell viability, which was above 90% for all vials. Cells were then diluted, plated for the respective assays in collagen-coated plates, and allowed to adhere for 3 h. Subsequently, cells were treated with the test compounds in INVITROGRO™ HI Medium (BioIVT, Brussels, BE) and incubated for 24 h. Collagen type I (rat) was purchased at Corning (New York, USA) and prepared as described (Haas et al. 2023b).

### Isolation of primary rat hepatocytes

Male Wistar rats (6–8 weeks old) were obtained from Janvier Labs (Le Genest-Saint-Isle, France). The rats were kept at the RPTU animal facility in accordance with the GV-Solas recommendations for rodent housing (light cycle, temperature, humidity, cages, etc.) and the specified pathogen-free (SPF) hygiene concept. The rats received a standard diet ad libitum and were acclimatized for at least one week before they were used for the isolation of primary hepatocytes. To this end, the animals were anaesthetised by i.p. administration of pentobarbital. Primary rat hepatocytes (PRH) were then isolated by two-step EGTA/collagenase perfusion as described previously (Carlsson and Fahrer 2023). The cell viability was assessed by trypan blue exclusion and was always above 90%. The cells were then seeded in collagen-coated 24-well-plates, allowed to attach for 3 h and afterwards treated with ES (0–2000 µM) or 1′OH-ES (0–250 µM) for 24 h.

### Chemicals and cell treatments

ES was purchased from PhytoLab (Vestenbergsreuth, Germany) and dissolved in dimethyl sulfoxide (DMSO) (Fisher Scientific, Hampton NH, USA) at a stock concentration of 2 M. The stock solution was then diluted in cell culture medium to reach the final test concentration, containing 0.1% DMSO as solvent at every treatment. The test concentrations of ES and 1’-OH-ES were chosen based on previous work (Schulte-Hubbert et al. 2020; Yang et al. 2020) and in accordance with the OECD recommendation for genotoxicity testing (OECD 2023), with respect to cytotoxicity, solubility and solvent concentration. 1′OH-ES was synthesised as described before (Cartus et al. 2012). In brief, *p*-anisaldehyde was dissolved in dry tetrahydrofuran under anhydrous conditions. Vinyl magnesium bromide was added and the solution was heated at 50 °C for 90 min. The crude 1′OH-ES solution was then purified by column chromatography. The yield was 42% and the identity was confirmed by ^1^H-NMR as well as ^1^H-^1^H-COSY-NMR (supporting information, Fig. 1A, B). Depending on the endpoint, various positive controls were used, including tert-butyl hydroperoxide (tBuOOH; Sigma-Aldrich, St. Louis MO, USA), etoposide (Hycultec, Beutelsbach, Germany), mitomycin C (MMC; Sigma-Aldrich, St. Louis MO, USA), cisplatin (Hycultec, Beutelsbach, Germany) and saponin (Serva, Heidelberg, Germany).

### DNA isolation, hydrolysis and quantification of DNA adducts

To this end, 6 × 10^6^ cells were seeded per 10 cm dish and allowed to attach overnight. Cells were then treated for 24 h with ES (0–2000 µM) or 1′OH-ES (0–35 µM), harvested including the supernatant, and centrifuged at 300 *g* for 5 min. The pellets were washed with phosphate-buffered saline (PBS) and stored at – 20 °C until further use. DNA was isolated as described previously (Carlsson et al. 2022) and DNA concentration as well as purity were determined with the NanoDrop ND1000 photometer (Thermo Scientific, Waltham MA, USA). As a next step, 30 µg DNA of each sample was digested overnight at 37 °C with micrococcal nuclease (Worthington, Lakewood NJ, USA) and phosphodiesterase II (Worthington, Lakewood NJ, USA) as reported (Carlsson et al. 2022). Afterwards ^15^*N*-E3′-*N*^2^-dG (2 nM), ^15^*N*-E3′-*N*^6^-dA (0.5 nM) as well as ^15^*N*-dG (20 µM) (Silantes, München, Germany) were added as internal standards. The isotope-labelled internal DNA adduct standards and external DNA adduct standard were synthesised as previously described (Schumacher et al. 2013). Subsequently, the samples were incubated overnight at 37 °C with alkaline phosphatase (Sigma-Aldrich, St. Louis MO, USA), followed by precipitation of remaining proteins with ethanol and centrifugation at 20,800 *g*. The supernatant was narrowed down in a vacuum centrifuge (Eppendorf concentrator plus, Eppendorf, Hamburg, Germany) at 60 °C and 1400 *g* for 2–4 h and the resulting pellet was dissolved in 75% methanol. After another centrifugation step, the solution was transferred into glass vials with inserts.

The DNA adducts were measured via UHPLC–MS/MS with a multiple reaction method (MRM) using a UHPLC system (Agilent 1290 infinity; Agilent Technologies, Santa Clara CA, USA) consisting of a binary pump (G4220A), an autosampler (G4226A) and a column oven (G1316C). The eluent consisted of 0.1% acetic acid and UHPLC grade methanol. Injection volume was 5 μL and the flow rate was 0.2 mL/min. The gradients are shown in the supporting information, Fig. 2A, B. As a separation column, a Waters XTerraMS C18 2.5 µm (2.1 × 50 mm) was used at 35 °C. The UHPLC was coupled to the Sciex QTrap 5500 MS using a declustering potential of 91 V, an entrance potential of 10 V, a collision energy of 33 V, a collision cell entrance potential of 10 V, curtain gas of 25 psi, an ion spray voltage of 5500 V, a temperature of 550 °C, and an ion source gas of 35 psi as specific mass spectrometry parameters. Measurement was performed with an electron spray ionisation in positive mode using MRM to quantify both adducts. For further quantification of the adduct levels, a standard curve of both adducts was carried along ranging from 60 to 100,000 pM, also containing isotope-labelled standards for reference. Furthermore, the dG-content of each sample was determined via LC–MS/MS with stable isotope dilution analysis to normalise the adduct levels to the overall nucleoside content (Carlsson et al. 2022; Stegmüller et al. 2018). The limit of detection (LOD) and the limit of quantification (LOQ) for E3′-*N*^2^-dG and E3′-*N*^6^-dA are provided in supplementary Table S1. The specific compound parameters for both methods are provided in supplementary Table S2.

### Confocal immunofluorescence analysis

Cells were seeded in 12-well slides with a removable grid (ibidi, Gräfelfing, Germany) at a density of 1.5 × 10^4^ cells per well and allowed to attach for 24 h. Analysis of CYP1A2 expression by confocal immunofluorescence microscopy was performed as described (Haas et al. 2023a; Mimmler et al. 2016). Cells were fixed with 4% paraformaldehyde, washed three times with phosphate-buffered saline (PBS), and blocked with 5% bovine serum albumin (BSA) in PBS/0.3% Triton X-100. Subsequently, the cells were incubated with a primary antibody against CYP1A2 (see supplementary Table S3) overnight at 4 °C. Afterwards, the cells were washed several times with PBS and PBS/0.4 M NaCl before incubation with a secondary antibody labelled with AlexaFluor 488 (1:400 in 5%-BSA in PBS/ 0.3%-Triton-X-100) for 1.5 h at room temperature (see supplementary Table S3). Cells were washed and embedded with Vectashield^®^ containing DAPI (Vector Labs, Burlingame, CA, USA). The samples were analysed with a Zeiss Axio Observer 7 microscope with a 10 × air objective (plan-apochromat 10x/0,45 M27) and the LSM 900 confocal laser scanner, whereas the pictures were processed with Zen Software 3.4 (Carl Zeiss Microscopy, Jena, Germany).

### Assessment of DNA strand breaks via the alkaline comet assay.

Cells were seeded at a density of 5 × 10^5^ cells in 3.5 cm dishes, left to adhere overnight, and treated for 24 h with ES (0–2000 µM) or 1′OH-ES (0–35 µM). As a positive control, tBuOOH (20 min, 200 µM) was used. Subsequently, the alkaline comet assay was performed to quantify DNA strand breaks as previously described (Haas et al. 2023b). Per sample, at least 50 comets were counted with Comet IV software (Instem, Staffordshire, UK) using a Zeiss Axio Observer 7 microscope equipped with an Axiocam 305 mono and ZEN 3.2 software (Carl Zeiss, Jena, Germany).

### SDS-PAGE and western blot analysis

γH2AX and p53 levels were assessed by SDS-PAGE and western blotting as reported (Fahrer et al. 2013). To this end, 5 × 10^5^ cells were seeded in 3.5-cm dishes to adhere overnight. The cells were then treated for 24 h with ES (0–2000 µM) or 1′OH-ES (0–50 µM) and subsequently harvested in 1 × Laemmli buffer containing 40 mM Tris–HCl, pH 6.8, 1.6% SDS, 8% glycerol, 0.016% bromphenol blue, and 0.8% β-mercaptoethanol. Etoposide and cisplatin were used as positive controls. Proteins were separated by SDS-PAGE followed by the transfer onto a nitrocellulose membrane using the wet blot method. The successful transfer was confirmed by Ponceau S staining of the membrane before blocking with 5% non-fat dry milk in Tris-buffered saline with Tween-20 (MP-TBS-T). Subsequently, the membrane was incubated with the respective primary antibodies overnight at 4 °C. The membranes were washed three times for 5 min with TBS-T before incubating with the respective secondary antibody coupled with horseradish peroxidase. The detection was carried out using the Western Lightning Plus-ECL reagent (Perkin Elmer, Waltham MA, USA) with the c300 chemiluminescence imager (Azure Biosystems, Dublin CA, USA). The used primary and secondary antibodies are detailed in supplementary Table S3.

### Real-time PCR

Gene expression analysis was essentially performed as described previously (Christmann et al. 2016). To quantify the mRNA expression level of *CYP1A2*, *SULT1A1*, and *SULT1C2*, total RNA from HepG2, HepG2-CYP1A2, and PRH (5 × 10^6^ cells each) was isolated using the NucleoSpin^®^ RNA Kit from Macherey–Nagel (Düren, Germany). 0.5 µg RNA was then transcribed into cDNA using the Verso cDNA Kit (Thermo Scientific, Dreieich, Germany) according to the manufacturer’s protocol. PCR was performed using the CFX96 Real-Time PCR Detection System (Biorad, München, Germany) and the GoTaq^®^ qPCR Master Mix (Promega, Madison, USA). Analysis was performed using the CFX Manager^™^ software. The used primers are shown in supplementary Table S4.

### Detection of cytotoxicity via the resazurin reduction assay

HepG2 cells were seeded in 96-well-plates (24 h 4.5 × 10^4^ per well, 72 h 1.5 × 10^5^ per well), grown overnight, and treated with ES (0–2000 µM) or 1′OH-ES (0–250 µM). DMSO was used as solvent control, while etoposide and saponin served as positive controls. PHH were seeded in 96-well plates precoated with collagen at a cell density of 7 × 10^4^/well, left to adhere for 3 h, and treated as described above for 24 h. After treatment, medium was discarded and replaced by resazurin reaction solution containing 44 µM resazurin in NaCl/Pi buffer (1.1 mM KH_2_PO_4_, 154 mM NaCl, and 3.7 mM Na_2_HPO_4_∙H_2_O) diluted 1:10 in medium without FCS and P/S. The cells were then incubated for 1.5 h and fluorescence was measured using a Spark^®^ microplate reader (Tecan, Männedorf, Switzerland) with an excitation at 544 nm and emission at 590 nm. The cell viability was calculated relative to the negative control (set to 100%). The effective concentration, which reduced cell viability by 50% (EC_50_), was determined with GraphPad Prism software (Version 8) by fitting the data with a sigmoidal, non-linear function as reported (Dörsam et al. 2015).

### Flow cytometry-based micronucleus assay

The flow cytometry-based micronucleus assay was essentially performed as described (Bryce et al. 2007), with slight modifications. HepG2 and HepG2-CYP1A2 cells were seeded in 6 cm dishes each with 4 × 10^5^ cells and allowed to attach for 24 h. Cells were then treated for 24 h with ES (0–2000 µM) or 1′OH-ES (0–50 µM). DMSO served as solvent control, while MMC was used as a positive control. Subsequently, the medium was aspirated, and the cells were rinsed with PBS and incubated in fresh medium for another 72 h, which corresponds to 1.5–2 cell cycles. Following the recovery time, the cells were detached, harvested by centrifugation (300 g, 5 min), and washed with PBS. The nuclei of dead cells were stained via ethidium bromide monoazide (EMA) (Biotium, Fremont CA, USA) by resuspending the cell pellet in 125 µg/mL EMA in PBS with 2% FCS. The fluorescent dye was then activated by photolysis (60 W lightbulb, 30 cm spacing) for 20 min before addition of 9 mL PBS to remove unbound dye. The cells were then lysed for 1 h in buffer containing 0.584 mg/mL NaCl, 1 mg/mL sodium citrate, 0.3 µL/mL NP40, 1 mg/mL RNAse A, as well as 0.2 µM of SytoxGreen (Thermo Fisher Scientific, Waltham MA, USA), a DNA-intercalating dye. Afterwards, the second lysis buffer containing 85.6 mg/mL sucrose, 15 mg/mL citric acid and 0.2 µM SytoxGreen was added. The samples were then incubated overnight at 4°C and measured with BD FACSCanto using a blue laser (488 nm) with PI (585/42 nm) and FITC filter (530/30 nm). 1.5 × 10^4^ cells were analysed using the BD FACSDiva software (BD Biosciences, Heidelberg, Germany). A schematic overview of the gating strategy is provided in the supporting information, Fig. 3.

### Cytokinesis-block micronucleus assay (CBMN)

The CBMN assay was performed according to the OECD test guideline #487. Therefore, 2 × 10^5^ cells were seeded in 3.5 cm dishes and allowed to adhere for 24 h**.** The cells were then treated with ES (0–2000 µM) or 1′OH-ES (0–50 µM). DMSO and MMC were included as solvent and positive controls, respectively. After the 24 h treatment, the medium was discarded and the cells were incubated with fresh medium, containing 6 µg/mL cytochalasin B as cytokinesis inhibitor. The cells were then left to grow for 72 h, allowing for 1.5–2 × cell cycles. Subsequently, the cells were rinsed with PBS and fixed with ice-cold methanol for 10 min at –20 °C. Cells were washed and embedded with Vectashield® containing DAPI (Vector Labs, Burlingame, CA, USA). The samples were analysed with a Zeiss Axio Observer 7 microscope equipped with an Axiocam 305 mono and a 63 × oil immersion objective (plan-apochromat 63x/1.40 DIC M27) using ZEN Software 3.2. 500 binucleated cells per sample were analysed for micronuclei formation.

### BMC modelling of the dose–response curves

To assess the genotoxic and mutagenic potency of ES and its metabolite 1′OH-ES, a benchmark concentration (BMC) modelling was performed with the data sets using the PROAST software (EFSA) followed by model averaging of the best four curve fits (supplementary Table S5) as described before (Haas et al. 2023b). As benchmark response (BMR) for all concentration–response curves (adducts, micronuclei), a 2.0-fold increase vs. solvent control was used (BMC_100_, critical effect size = 1). The determined BMC_100_ and the corresponding upper and lower boundary (BMCU and BMCL, respectively) of the 90% confidence interval (CI) were used for potency comparison.

### Ethics

All animal experiments were approved by the government of Rhineland-Palatinate and the Animal Care and Use Committee of the RPTU in Kaiserslautern, Germany (# 23177-07/G 22-2-028). The experiments were performed in accordance with the German Federal Law and the guidelines for the protection of animals.

### Statistics

Experiments were performed independently at least three times, except where otherwise stated. Results from representative experiments are shown. Values underwent Grubbs’ test to exclude outliers and are displayed as mean, and error bars represent standard error of the mean (SEM) using the GraphPad Prism 8.0 Software (GraphPad Software Inc.). Statistical analysis was performed using two-sided Student’s t test and statistical significance was defined as *p* < 0.05.

## Results

### Quantification of E3′-*N*^2^-dG and E3′-*N*^6^-dA adduct formation after treatment with ES or 1′OH-ES

**Fig. 1:**
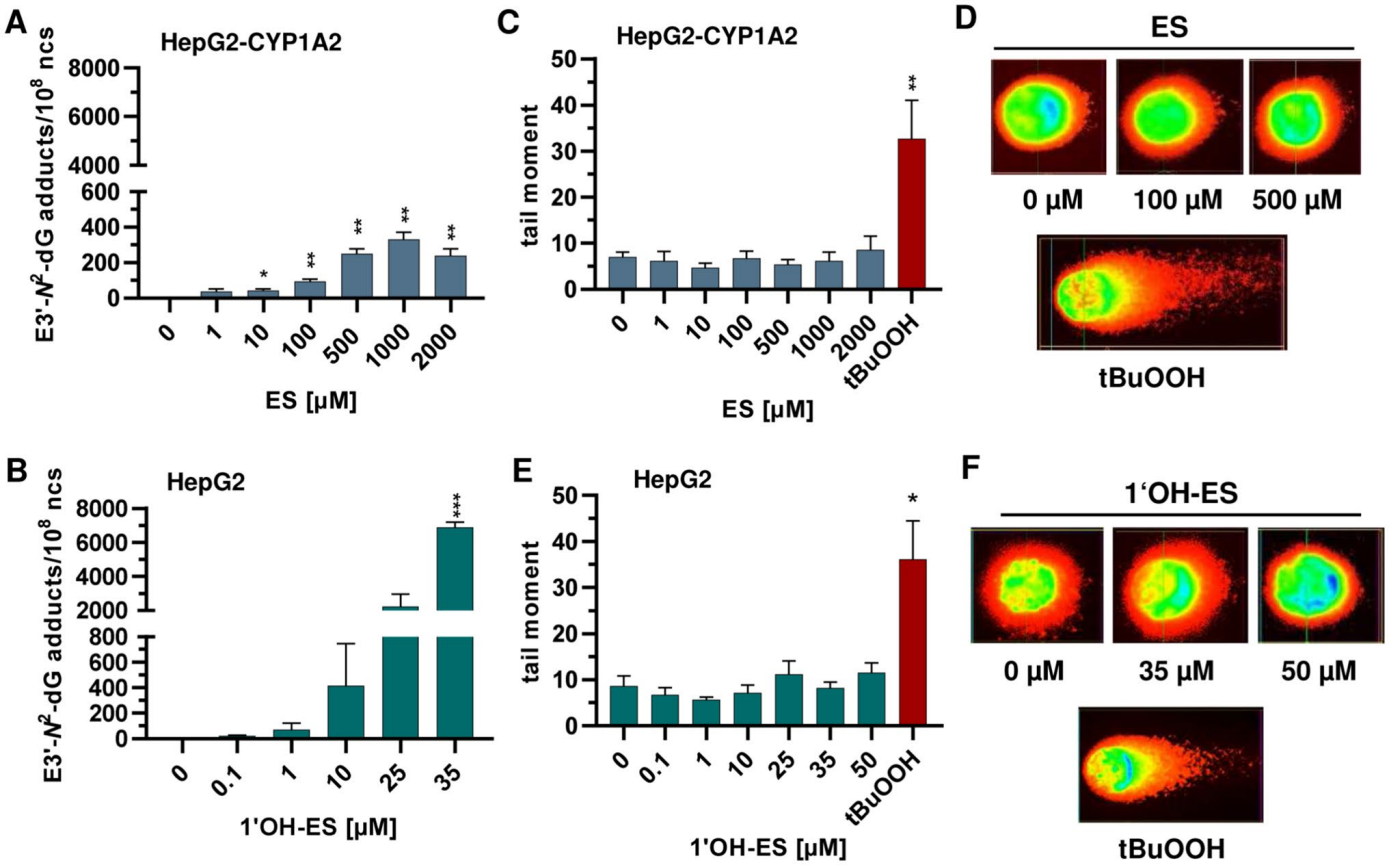
DNA adduct formation and DNA strand break induction in HepG2-CYP1A2 and HepG2 cells after estragole and 1′-hydroxyestragole treatment.** A** Quantification of E3′-*N*^2^-dG adducts in HepG2-CYP1A2 after treatment with up to 2 mM estragole (ES) for 24 h (*n* = 4). **B** Quantification of E3′-*N*^2^-dG adducts in HepG2 cells after treatment with up to 50 µM 1′-hydroxyestragole (1′OH-ES) for 24 h (*n* = 2–4). **C** DNA strand breaks determined via the alkaline comet assay after treatment of HepG2-CYP1A2 cells with ES for 24 h (n ≥ 3). **D** Representative comet pictures. *Tert*-Butylhydroperoxide (tBuOOH) served as positive control (200 µM, 20 min). **E** DNA strand breaks determined via the alkaline comet assay after treatment of HepG2 cells with 1′OH-ES for 24 h (n ≥ 4). **F** Representative comet pictures. tBuOOH (200 µM, 20 min) was used as positive control. DMSO was used as solvent control. All data given as mean + SEM. Statistical analysis was performed using two-tailed, unpaired t test versus the LOQ (**A**, **B**) or the solvent control (**C**, **E**) (*p* > 0.05, *p < 0.05, **p < 0.01, ***p < 0.001)

To study possible genotoxic and mutagenic effects of ES, a suitable human liver cell model with metabolic competence is required. Therefore, we used human HepG2 cells stably transduced with cDNA of human *CYP1A2* (Steinbrecht et al. 2020) and re-analysed mRNA as well as protein expression of both CYP1A2 and SULT1A1. As expected, parental HepG2 showed a low *CYP1A2* expression, whereas stably transduced HepG2-CYP1A2 and primary rat hepatocytes (PRH) displayed high *CYP1A2* expression levels (supporting information, Fig. 4A). Importantly, both HepG2 liver models showed comparable *SULT1A1* and *SULT1C2* levels, which were, however, below those observed in PRH (supporting information, Fig. 4A). These findings were also reflected on the protein level as revealed by western blot analysis and further validated in HepG2-CYP1A2 using confocal microscopy (supporting information, Fig. 4B, C). As a next step, we investigated the ES and 1′OH-ES-derived DNA adduct formation via ultra-high performance liquid chromatography (UHPLC) coupled with mass spectrometry (MS/MS). HepG2-CYP1A2 cells were treated with up to 2 mM ES for 24 h, revealing a concentration-dependent formation of the main DNA adduct E3′-*N*^2^-dG (Fig. 1A). Already at 1 µM ES, 37 adducts/10^8^ nucleosides (ncs) were measured, which further increased up to a maximum level at 1 mM ES with 332 adducts/10^8^ ncs (Fig. 1A). In the highest treatment group (2 mM ES) a moderate decline was observed. It should be mentioned that the minor DNA adduct, E3′-*N*^6^-dA, was neither quantifiable nor detectable at any tested ES concentration. Exposure of parental HepG2 cells to the proximal carcinogen 1′OH-ES for 24 h also led to a concentration-dependent generation of E3′-*N*^2^-dG adducts, however, with 10–50-fold higher E3′-*N*^2^-dG levels than those observed after equimolar ES concentrations (Fig. 1B). At the lowest test concentration of 1′OH-ES (0.1 µM), 24 adducts/10^8^ ncs were quantified. For the highest test concentrations (25 and 35 µM), 2237 and 6881 E3′-*N*^2^-dG adducts/10^8^ ncs were obtained, respectively. In contrast to ES treatment, 1′OH-ES also caused E3′-*N*^6^-dA adducts at the two highest concentrations tested, amounting to 64 and 139 E3′-*N*^6^-dA adducts/10^8^ ncs, respectively (supporting information, Fig. 5A). In summary, our results showed a concentration-dependent increase of E3′-*N*^2^-dG adducts after exposure to both ES and 1′OH-ES, however, with 10–50-fold higher adduct yield for the phase I-metabolite.

### Assessment of DNA strand break induction by ES or 1′OH-ES via the alkaline comet assay

To determine whether ES and 1′OH-ES cause DNA strand breaks, an alkaline comet assay was performed, which allows the quantification of both DNA single- and double-strand breaks as well as alkali-labile sites (Azqueta and Collins 2013). HepG2-CYP1A2 cells were treated with concentrations up to 2 mM ES for 24 h, which did not elicit an increase of DNA strand break levels. Only at the highest ES concentration (2 mM), a small but not statistically significant increase was detected as compared to the solvent control (Fig. 1C, D). The phase I-metabolite 1′OH-ES also showed no clear DNA strand break induction in HepG2 cells after 24 h (Fig. 1E, F). At 50 µM 1′OH-ES, a weak increase was observed as compared to the solvent control, which is in line with the representative microscopic pictures (Fig. 1F). As positive control, the peroxide tBuOOH was used, which causes oxidative DNA damage and DNA strand breaks (Wenz et al. 2019). Importantly, tBuOOH showed a strong DNA strand break induction in both HepG2 cell models. In conclusion, both ES and 1′OH-ES treatment caused no DNA strand breaks after the 24 h treatment despite the concentration-dependent formation of DNA adducts.

### Analysis of the DDR markers p53 and γH2AX following incubation with ES or 1′OH-ES

**Fig. 2:**
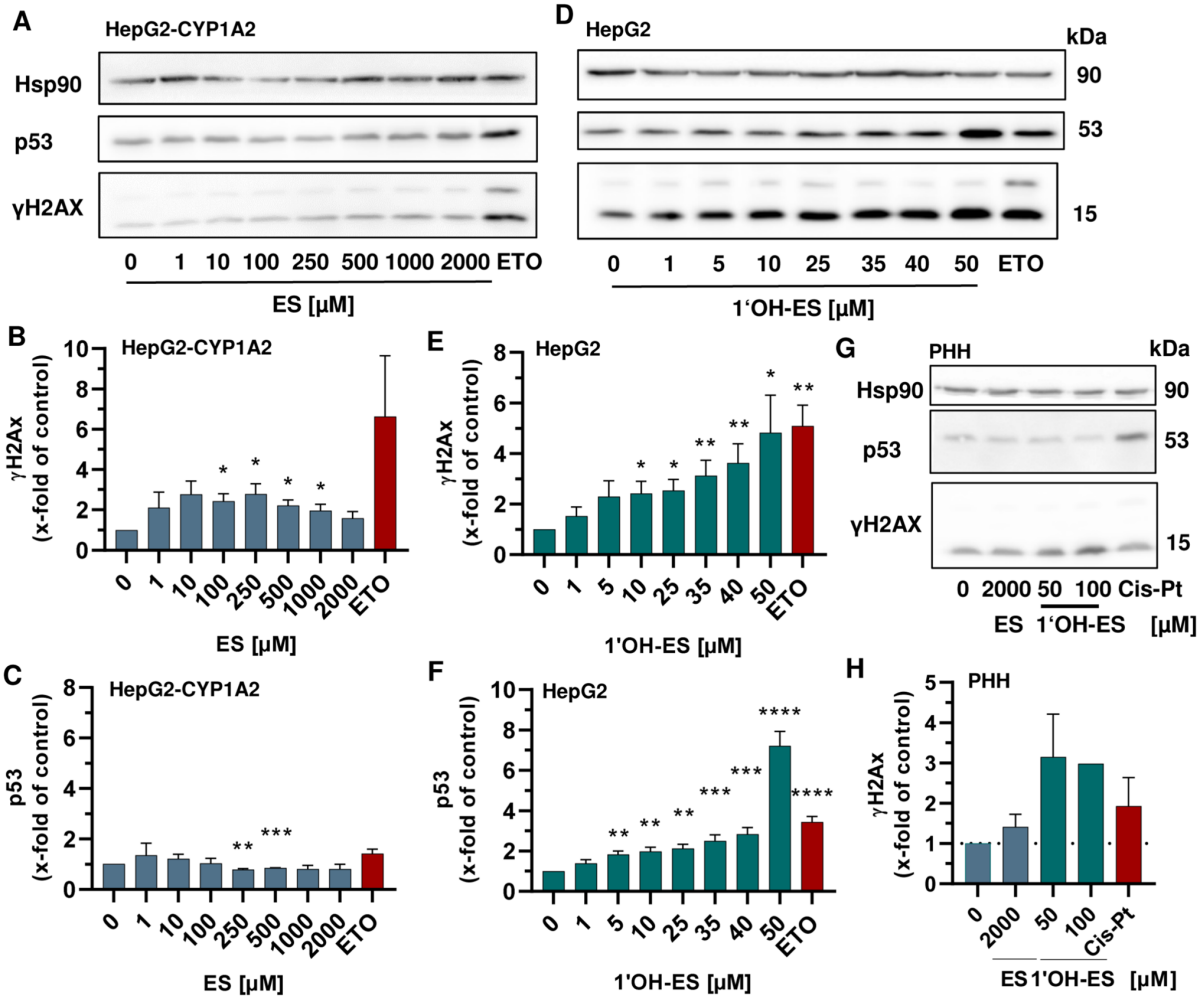
Analysis of γH2AX levels and p53 accumulation in HepG2 liver cell models and PHH treated with estragole and 1′-hydroxyestragole.** A–C** Representative Western blot experiments (**A**) and densitometric evaluation of yH2AX (**B**) and p53 (**C**) after treatment of HepG2-CYP1A2 with estragole (ES) for 24 h (*n* = 3)*.* **D–F** Representative Western blot experiments (**D**) and densitometric evaluation of yH2AX (**E**) and p53 (**F**) after treatment of HepG2 with 1′-hydroxyestragole (1′OH-ES) for 24 h (*n* ≥ 3)*.* **G–H** Representative Western blot experiments (**G**) and densitometric evaluation of yH2AX (**H**) after treatment of PHH with ES and 1′OH-ES for 24 h (*n* ≥ 2). Hsp90 served as loading control. DMSO was used as solvent control, while etoposide (ETO) and cisplatin (Cis-Pt) served as positive controls. All data given as mean + SEM. Statistical analysis was performed using two-tailed, unpaired t test versus the solvent control (*p < 0.05, **p < 0.01, ***p < 0.001, ****p < 0.0001)

To study the DNA damage response (DDR) to ES and 1′OH-ES-derived DNA adducts, γH2AX and p53 were analysed as established genotoxicity markers (Haas et al. 2023b; Nikolova et al. 2014). HepG2-CYP1A2 cells were treated with ES for 24 h, collected, and subjected to western blot analysis using Hsp90 as a loading control. γH2AX showed a moderate, concentration-dependent increase up to 250 µM ES (threefold vs. control) followed by a decline at higher concentrations (Fig. 2A, B). As positive control, the topoisomerase II inhibitor and anticancer drug etoposide was used that gives rise to DNA double-strand breaks (Li et al. 2014; Montecucco et al. 2015). As expected, etoposide caused a strong γH2AX induction (6.7-fold vs. control). No p53 accumulation was observed following ES exposure, whereas the positive control etoposide displayed a moderate p53 increase (Fig. 2A, C). In contrast to these findings, the phase I- metabolite 1′OH-ES caused a clear, concentration-dependent induction of both γH2AX and p53 in HepG2 cells (Fig. 2D–F). Already at a concentration of 5 µM 1′OH-ES, a 2.2-fold increase in γH2AX was observed, culminating in a 4.8-fold induction at the highest test concentration (50 µM) (Fig. 2E). In line with these results, a concentration-dependent accumulation of p53 was observed, revealing a sevenfold induction at 50 µM 1′OH-ES (Fig. 2F). As expected, the positive control etoposide caused both γH2AX formation and p53 accumulation (Fig. 2E, F). Finally, both genotoxicity markers were assessed in PHH, which were challenged with selected concentrations of both ES and 1′OH-ES, followed by SDS-PAGE and western blot detection. ES treatment resulted in a moderate induction of γH2AX (1.4-fold vs. control), but did not cause p53 accumulation (Fig. 2G, H). The metabolite 1′OH-ES induced robust γH2AX formation (threefold vs. control) (Fig. 2H), whereas p53 levels even slightly declined (supporting information, Fig. 5B). In non-proliferating PHH, the antineoplastic drug and DNA crosslinker cisplatin (Kelland 2007) served as a positive control, as it had already been used successfully in a previous study with PHH (Haas et al. 2023b). Cisplatin caused both γH2AX and p53 induction. Taken together, the metabolite 1′OH-ES increased γH2AX formation in a concentration-dependent manner in both HepG2 and PHH, whereas the mother compound ES showed only moderate effects. Interestingly, a strong p53 accumulation was observed following 1′OH-ES treatment in HepG2 cells, whereas in PHH p53 levels decreased.

### Analysis of cytotoxic effects following exposure to ES or 1′OH-ES.

**Fig. 3:**
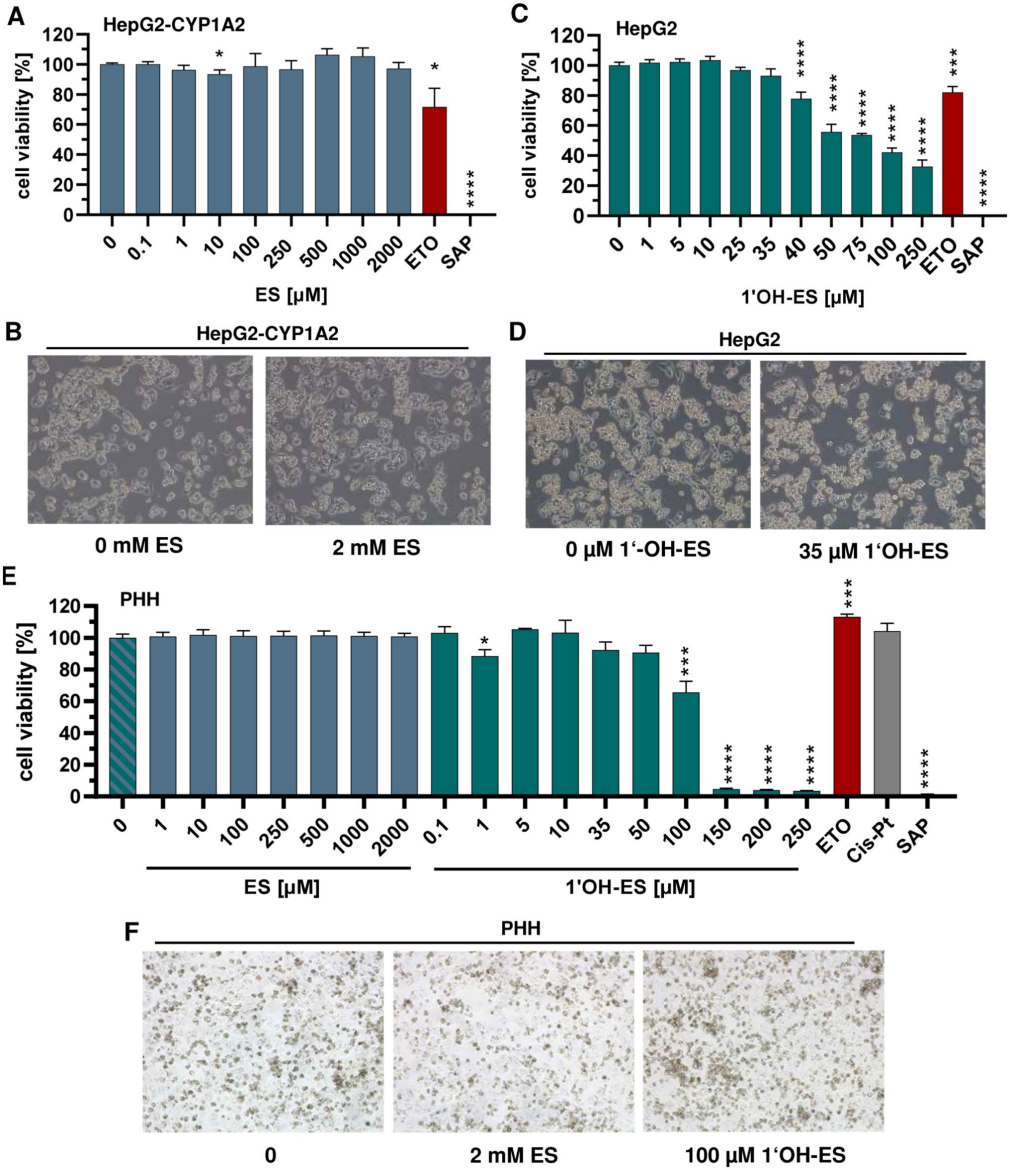
Cytotoxic effects of estragole and 1′-hydroxyestragole in HepG2 liver cell models and PHH after 24 h.** A** Cell viability of HepG2-CYP1A2 after treatment with estragole (ES) over 24 h (*n* = 3). **B** Representative images of treated HepG2-CYP1A2 (10× magnification). **C** Cell viability of HepG2 after treatment with 1′-hydroxyestragole (1′OH-ES) over 24 h (*n* ≥ 3). **D** Representative images of treated HepG2 (10× magnification). **E** PHH exposed to ES (*n* = 3) and 1′OH-ES (*n* ≥ 2) over 24 h. **F** Representative images of treated PHH (10× magnification). Saponin (SAP, 0.1%) and etoposide (ETO, 10 µM) served as positive controls, whereas DMSO was used as solvent control. All data given as mean + SEM. Statistical analysis was performed using two-tailed, unpaired t test versus the solvent control (*p < 0.05, ***p < 0.001, ****p < 0.0001)

The resazurin reduction assay was used to study the cytotoxicity of ES and 1′OH-ES after 24 h and 72 h, respectively. The later time point (72 h) was selected to consider replication-dependent effects of ES-derived DNA damage. The parent compound ES showed no cytotoxicity in HepG2-CYP1A2 cells neither after 24 h (Fig. 3A) nor after 72 h (supporting information, Fig. 6A), which was in line with the absence of morphological changes (Fig. 3B and supporting information, Fig. 6B). On the contrary, the metabolite 1′OH-ES induced time- and concentration-dependent cytotoxic effects in HepG2 cells (Fig. 3C and supporting information, Fig. 6C). This is illustrated by an EC_50_ value of 43 µM after 24 h (supporting information, Fig. 7A), which decreased to 27 µM after 72 h (supporting information, Fig. 7B). On the morphological level, effects were hardly visible after treatment with 35 µM 1′OH-ES for 24 h as compared to the negative control (Fig. 3D). In contrast, cytotoxicity of 1′OH-ES was clearly seen after 72 h as reflected by the reduced cell number as well as the increased frequency of round and detached cells (supporting information, Fig. 6D). In support of these results, 1′OH-ES was also cytotoxic in PHH, however, with a higher EC_50_ value of 107 µM (24 h, Fig. 3E and supporting information, Fig. 7C). It should be noted that no overt cytotoxicity was observed at the morphological level upon incubation with 100 µM 1′OH-ES (Fig. 3F). Consistent with the findings in HepG2-CYP1A2 cells, the mother compound ES did not affect viability in PHH even at test concentrations up to 5 mM (Fig. 3E, F). The positive control etoposide was cytotoxic in both HepG2 cell models in a time-dependent manner, whereas no effect was seen in PHH (Fig. 3A, C, E). Finally, cytotoxicity was assessed in PRH, which were very sensitive towards 1′OH-ES with an EC_50_ value of 23 µM (supporting information, Figs. 6G and 7D). Furthermore, a slight cytotoxic effect was revealed in PRH for the mother compound ES at 2 mM (supporting information, Fig. 6E, F). In summary, ES was not cytotoxic at all in human liver cell models, whereas 1′OH-ES triggered cytotoxicity in a time- and concentration-dependent manner.

### Analysis of the micronucleus formation after treatment with ES or 1′OH-ES.

**Fig. 4:**
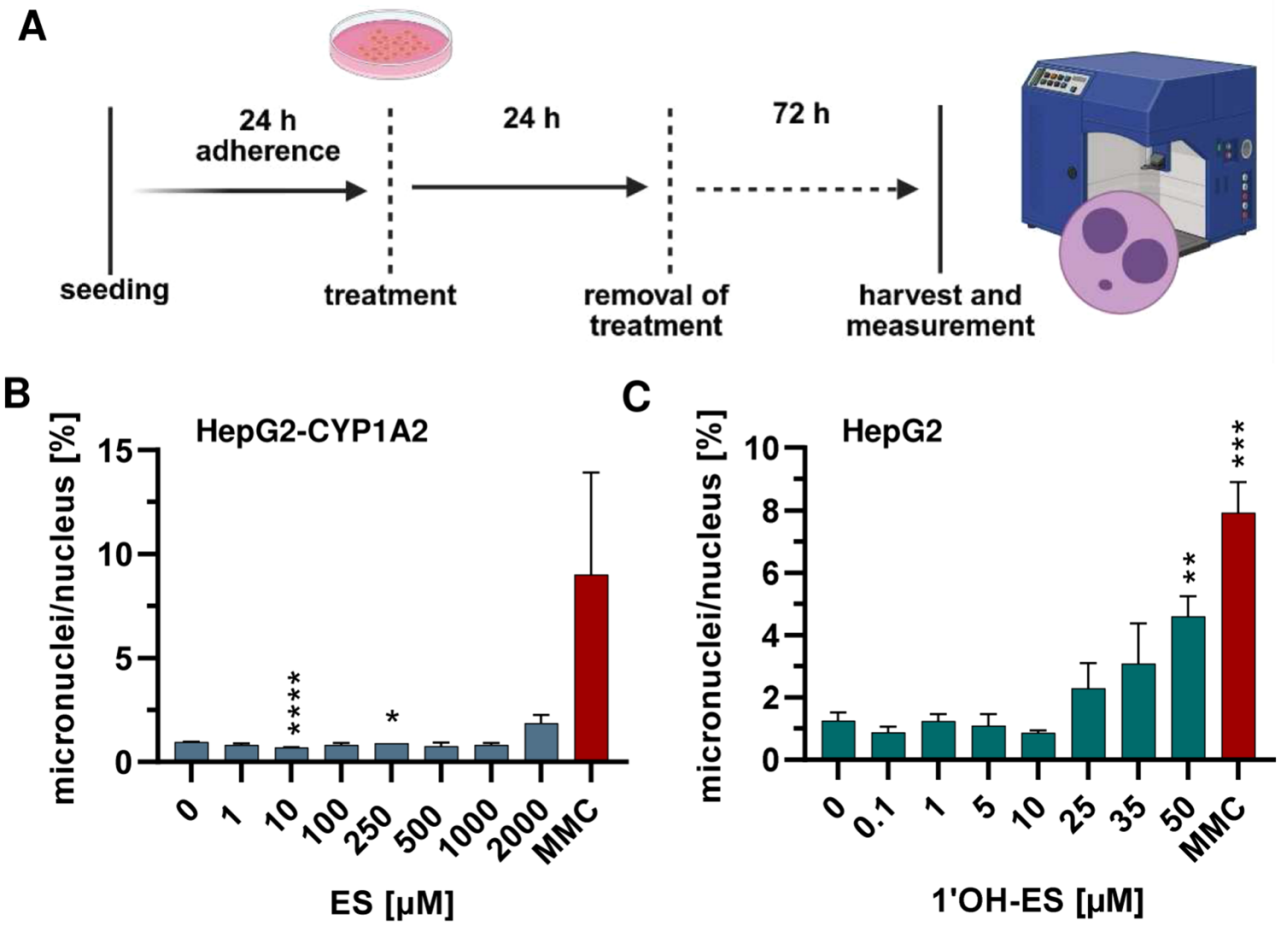
Assessment of the clastogenic potential of estragole and 1′-hydroxyestragole in HepG2 and HepG2-CYP1A2 cells.** A** Experimental setup to analyse micronuclei (MN) formation, cartoon created in BioRender. Fahrer, J. (2025) https://BioRender.com/p78b810**B** Assessment of MN induction in HepG2-CYP1A2 cells after treatment with estragole (ES) over 24 h with a 72 h recovery time (*n* ≥ 3). **C** Assessment of MN induction in HepG2 cells after treatment with 1′-hydroxyestragole (1′OH-ES) over 24 h with a 72 h recovery time (*n* ≥ 3). Mitomycin C (MMC, 0.25 µM) served as positive control, while DMSO was used as solvent control. Micronuclei were determined by a flow cytometry-based method. All data given as mean + SEM. Statistical analysis was performed using two-tailed, unpaired t test versus the solvent control (*p < 0.05, **p < 0.01, ***p < 0.001, ****p < 0.0001)

Subsequently, we determined the formation of micronuclei (MN) as an established marker of genomic instability (Fahrer 2024). Therefore, a flow cytometry-based approach was used as reported previously (Bryce et al. 2007). According to the OECD test guideline #487, only concentrations with a cytotoxicity below 55 ± 5% should be used to assess MN induction (OECD 2023). We thus performed a resazurin reduction assay, in which the cells were treated for 24 h with ES or 1′OH-ES followed by a recovery period of 72 h corresponding to 1.5–2 cell cycles (Fig. 4A, supporting information, Fig. 8A, B). This setup was then used to determine MN formation, revealing a moderate clastogenic effect of ES (1.6-fold increase vs. solvent control), which was, however, not statistically significant (Fig. 4B). As positive control, the DNA cross-linking agent MMC (Tomasz and Palom 1997) was used in accordance with the OECD test guideline #487 (OECD 2023). MMC showed a strong ninefold induction of MN vs. solvent control (Fig. 4B). In turn, the metabolite 1′OH-ES triggered MN formation in HepG2 cells in a concentration-dependent manner, with a twofold increase already detected at 25 µM and a 4.6-fold increase at 50 µM (Fig. 4C). It must be noted that even at the top concentration (50 µM 1′OH-ES), cell viability was above 70% in accordance with the OECD test guideline. As expected, MMC treatment caused a roughly eightfold increase in MN vs. solvent control (Fig. 4C). Importantly, the findings obtained by flow cytometry were confirmed via the microscopic cytokinesis-block micronucleus (CBMN) assay (supporting information, Fig. 8C, D), although a generally lower induction of MN by both ES and 1′OH-ES was observed. This could be attributable to the fact that only MN in binucleated cells were counted using the cytokinesis-inhibitor cytochalasin B. Both ES and 1′OH-ES induced a concentration-dependent increase, starting at 500 µM ES and 5 µM 1′OH-ES, respectively (supporting information, Fig. 8C, D). The top concentrations (2000 µM ES and 50 µM 1′OH-ES) caused roughly a twofold induction of MN as compared to solvent control. The positive control MMC showed a robust increase in MN as observed before using the flow cytometry-based method (supporting information, Fig. 8C, D). In conclusion, ES induced MN formation at concentrations of 1 mM and above, whereas the metabolite 1′OH-ES led to an increased MN frequency in the lower µM range.

### Molecular dosimetry of E3′-N^2^-dG adducts, clastogenicity and cytotoxicity as well as quantitative analysis by BMD modelling

**Fig. 5:**
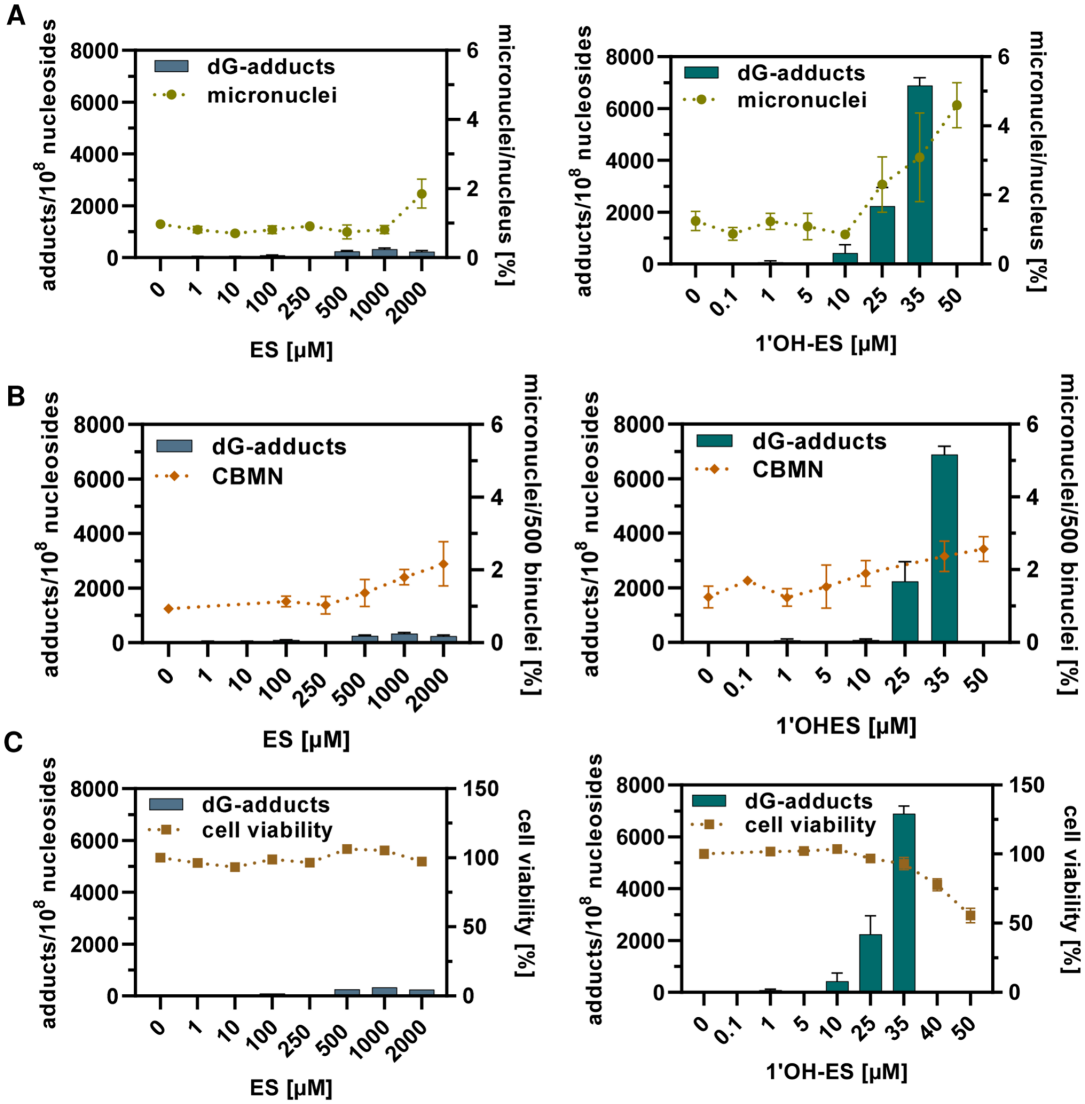
Molecular dosimetry of DNA adduct levels and their association with clastogenicity and cytotoxicity in HepG2 liver cell models.** A** E3′-*N*^2^-dG levels vs micronuclei determined by flow cytometry **B** E3′-*N*^2^-dG levels vs micronuclei determined by CBMN assay **C** E3′-*N*_2_-dG levels versus cytotoxicity determined by resazurin reduction assay. Estragole (ES) treatment of HepG2-CYP1A2 cells, 1′-hydroxyestragole (1′OH-ES) treatment of HepG2 cells. All data given as mean + SEM

We performed molecular dosimetry of the induced E3′-*N*^2^-dG adducts vs. the biological endpoints’ clastogenicity and cytotoxicity. First, we compared the E3′-*N*^2^-dG adduct levels with measured MN formation assessed by flow cytometry and the CBMN assay, respectively. For ES, an adduct level of 240–330 E3′-*N*^2^-dG adducts/10^8^ ncs caused a roughly twofold increase, whereas, for 1′OH-ES, a twofold increase was observed at an adduct level of 420–2237 adducts/10^8^ ncs (Fig. 5A, B). Next, we compared the E3′-*N*^2^-dG adduct levels with the cytotoxic effects induced by ES and 1′OH-ES. Only at the highest 1′OH-ES concentration, significant levels of cytotoxicity were observed, which corresponds to an E3′-*N*^2^-dG adduct level of 6881 adducts/10^8^ ncs (Fig. 5C). Remarkably, these adduct levels were by far not reached at any tested ES concentration. Taken together, this comparison illustrates that a certain E3′-*N*^2^-dG adduct level is required to trigger downstream biological effects, with the rank order MN formation > cytotoxicity.

To refine this approach and to compare the potency of ES and its phase I-metabolite 1′OH-ES, we used the concentration–response data for benchmark concentration (BMC) modelling with the PROAST software (supporting information, Figs. 9, 10). To this end, a critical effect size (CES) of one, i.e., a doubling of the measured DNA adduct or MN level as compared to controls, was used as benchmark response (BMR) with a confidence interval of 90%. With regard to DNA adduct formation, the determined critical effect concentration (CEC; represents the BMC) was 63.3 µM for ES and 2.7 µM for 1′OH-ES (Table 1). A BMC of 1116 µM was obtained for MN formation upon ES treatment using the CBMN method (Table 1). The data obtained with the flow cytometry-based method were not suitable for BMC modelling, since none of the concentrations tested caused an effect above the CES. As observed for the DNA adducts, the BMC for MN formation was much lower for 1′OH-ES (Table 1), consistently ranging between 30 and 32 µM depending on the used method (FCM vs. CBMN). Taken together, this analysis provided evidence for a 20–30-fold higher genotoxic and mutagenic potency of 1′OH-ES as compared to the parent compound ES. Moreover, it revealed that the BMCs for a clastogenic response to both ES and 1′OH-ES are 12–17-fold higher than the respective BMCs for DNA adduct formation, confirming that a certain level of DNA adducts must be present to cause clastogenicity.

**Table 1:** Benchmark concentration (BMC) values obtained by BMC modelling of concentration–response data (DNA adducts, MN formation) for ES and 1′OH-ES in human liver cells

	Endpoint	BMCL [µM]	BMC_100_ [µM]	BMCU [µM]
ES	E3′-*N*^2^-dG	18.9	63.3	104
	MN	555	1116	1755
1′OH-ES	E3′-*N*^2^-dG	0.75	2.65	5.65
	MN (FCM)	21.1	29.8	37.6
	MN	21.0	32.0	106

## Discussion

In the present work, we set out to analyse the concentration–response relationship for ES and its phase I-metabolite 1′OH-ES in human liver cell models, allowing for quantitative genotoxicity modelling, potency comparison, and molecular dosimetry of adduct formation in relation to clastogenicity and cytotoxicity. First, our experiments revealed 10–50-fold higher E3′-*N*^2^-dG adduct levels after treatment with 1′OH-ES as compared to the parent substance ES at equimolar concentrations. This is in agreement with a recent study, in which HepaRG cells and PRH were exposed to either 50 µM ES or 1′OH-ES, the latter causing 33–40-fold higher E3′-*N*^2^-dG levels (Yang et al. 2020). The same authors also provided evidence that ES failed to cause DNA adducts in parental HepG2 cells (Yang et al. 2020), highlighting the advantage of the metabolically competent HepG2-CYP1A2 cell model used in the present work. The minor DNA adduct E3′-*N*^6^-dA could only be quantified at higher 1′OH-ES concentrations in our study, with a ratio (E3′-*N*^6^-dA: E3′-*N*^2^-dG) of approximately 1:42. Interestingly, a ratio of 1:10 was reported in PRH following ES treatment (Schulte-Hubbert et al. 2020), which might indicate species-specific differences.

As a next step, DNA strand break induction by ES- and 1′OH-ES-derived DNA adducts was analysed using the alkaline comet assay. However, no or very low levels of DNA strand breaks were detected at all test concentrations for both ES and 1′OH-ES. This finding is in line with a previous study in HepaRG cells treated with up to 2 mM ES for 24 h, with a negative outcome (Le Hegarat et al. 2014). Furthermore, the structurally similar 1′-hydroxymethyleugenol (1′OH-ME) was negative in the alkaline comet assay as well (Carlsson et al. 2022). The lack of detectable DNA strand breaks might be attributable to the short half-life of DNA repair intermediates or a suboptimal sampling time (Ngo et al. 2021). Nevertheless, a concentration-dependent formation of γH2AX, a well-established marker for both DSBs and replication stress, was observed upon exposure to 1′OH-ES in HepG2 cells and PHH. This clearly indicates an activation of the DDR by ES-derived DNA adducts, likely mediated by the apical DDR kinases ATR and ATM as described for ME-derived DNA adducts (Carlsson et al. 2022). Consistent with this notion, we found a concentration-dependent accumulation of p53 after treatment with 1′OH-ES. On the other hand, no p53 induction was observed in PHH despite pronounced γH2AX formation. This might be due to different kinetics in p53 stabilisation or an altered p53 homeostasis in PHH. It should also be mentioned that ES failed to induce p53 in both HepG2 and PHH, which is likely attributable to the lower DNA adduct levels.

Furthermore, our experiments revealed clear cytotoxic effects for the metabolite 1′OH-ES in a concentration-dependent manner. The EC_50_ value for 1′OH-ES (43 µM) was in the range of that determined previously by Yang and colleagues (EC_50_ = 102 µM) using the MTT assay (Yang et al. 2022). The cytotoxicity of 1′OH-ES also increased in a time-dependent manner, indicating replication-dependent effects of ES-derived DNA damage. This is in line with findings for the structurally related 1’OH-ME (Carlsson et al. 2022; Groh and Esselen 2017). In contrast to this pronounced cytotoxicity of the metabolite, the mother compound ES did not impair cell viability even in the mM concentration range. One obvious explanation for the very low cytotoxic effect might be that ES is only partially converted to the proximal carcinogen 1′OH-ES, whilst at the same time, ES can undergo *O*-demethylation and subsequent glucuronidation as detoxification. The former reaction is also catalysed by CYP1A2 and was shown to occur as main pathway after exposure to ES (Anthony et al. 1987). In agreement with this finding, only a small proportion of ES was converted to the 1′OH-ES metabolite in human volunteers following oral uptake of ES via fennel tea or as encapsulated pure substance (Sangster et al. 1987; Zeller et al. 2009). Moreover, the DNA translesion polymerases κ and η have been shown to efficiently replicate in vitro over the E3′-*N*^2^-dG adduct (Deshmukh et al. 2023) as well as the structurally similar dA adduct derived from ME and safrole (Bagale et al. 2025), thereby avoiding replication stress-associated DDR and toxicity. Based on our data, more than 6000 E3′-*N*^2^-dG adducts/10^8^ ncs are required to impair viability and trigger cell death, which is only reached at 1′OH-ES concentrations ≥ 35 µM, but not by ES. In support of these findings, the structurally related 1′OH-ME was not cytotoxic at concentrations below 25 µM (Carlsson et al. 2022). Another aspect to consider is the stability of ES and 1′OH-ES. Due to the lack of reactive structural elements, both ES and 1′OH-ES are not prone to hydrolysis in solution, which is in contrast to the formed 1′-sulfooxy-metabolite that spontaneously decomposes (Punt et al. 2007). In our HepG2 test models with endogenous metabolic activation due to CYP1A2 and SULT1A1 expression, these reactions occur intracellularly. This is an important advantage, since negative or equivocal test results can be obtained for phenylpropenes in cell models requiring exogenous metabolic activation, such as V79 cells with S9-mix (Eisenreich et al. 2025).

Finally, we were able to demonstrate that both ES and its metabolite 1′OH-ES are clastogenic using the CBMN and MN flow assay. Importantly, this genotoxic effect was only observed at concentrations ≥ 1 mM ES and concentrations ≥ 25 µM 1′OH-ES in the human liver cell models. The observed clastogenicity of ES is in line with the previous reports using the MN assay without cytokinesis block in HepG2-CYP1A2 cells (Schulte-Hubbert et al. 2020) and with the CBMN assay performed in HepaRG cells (Le Hegarat et al. 2014). Interestingly, the latter study revealed 1 mM ES as the lowest effect concentration for MN formation, which is perfectly in line with our concentration–response data. Our results further provide evidence that a certain threshold adduct level, ranging between 240 and 2230 E3′-*N*^2^-dG adducts/10^8^ ncs, is required to trigger clastogenicity.

In our study, we dissected the molecular dosimetry of ES-derived DNA adducts with regard to different biological endpoints, such as DDR, clastogenicity and cytotoxicity. It should, however, pointed out that ES naturally occurs in the essential oil of fennel or basil together with other phenylpropenes, such as anethole and ME. Interestingly, anethole, an isomer of ES, was demonstrated to undergo the same metabolic activation pathway (Bergau et al. 2021; Monien et al. 2019). In vitro incubation of anethole with calf thymus DNA in the presence of rat S9-mix and the SULT co-substrate 3′-phosphoadenosine 5′-phosphosulfate revealed the formation of E3′-*N*2-dG adducts (Bergau et al. 2021). However, the adduct levels were several times lower than those caused by equimolar ES concentrations (Bergau et al. 2021), suggesting that anethole would contribute little to the total E3′-*N*2-dG adduct burden in a scenario of co-exposure. Furthermore, it is conceivable that phenylpropenes may compete for the same CYP isoenzyme, thus affecting the efficiency of their 1′-hydroxylation. In line with that assumption, co-incubation of equimolar combinations of ME and ES with Gentest microsomes expressing CYP1A2 showed a competitive interaction and revealed a strong attenuation of ES 1′-hydroxylation in the presence of ME (Jeurissen et al. 2007). A more recent study analysed the effects of combined exposure to ES and safrole in HepG2 cells using the respective 1′OH-metabolites (Yang et al. 2022). Here, additive cytotoxic effects were observed upon combined exposure, while the DNA adduct levels (E3′-*N*2-dG and S3′-*N*2-dG) were in a similar range independent of single or combined exposure (Yang et al. 2022). These findings indicate that there is no competitive interaction with regard to sulfo-conjugation catalysed by SULTs. Further studies are warranted to detail the mixture toxicity of phenylpropenes, particularly with regard to DNA adduct formation.

Another important aspect is the cellular fate of ES-derived DNA adducts. A recent publication provided evidence that E3′-*N*^2^-dG adducts accumulate in HepaRG cells following repetitive treatment with 50 µM ES for 4 days (Yang et al. 2021). The adduct accumulation very likely results from inefficient DNA repair as reported previously (Yang et al. 2020). The study revealed only partial removal of E3′-*N*^2^-dG adducts in wildtype CHO cells exposed to 50 µM 1′OH-ES, which was further attenuated in CHO cells deficient in nucleotide excision repair (Yang et al. 2020). These findings were corroborated in another work conducted in PRH that were treated with ES. There, a time-dependent increase in both E3′-*N*^2^-dG and E3′-*N*^6^-dA adducts was observed, which reached maximum levels after 6 h and subsequently declined by 20–30% after 24 h (Schulte-Hubbert et al. 2020). The reported DNA adduct persistence and accumulation over time is relevant in the setting of a ‘tolerable’ chronic ES exposure. In humans, the dietary ES uptake was estimated to amount to 0.01 mg/kg bw and day (Punt et al. 2016; Yang et al. 2021). Based on PBPK modelling, an ES concentration of 0.01 µM in liver venous blood could be predicted, which would give rise to 0.0048 E3′-*N*^2^-dG adducts per 10^8^ nucleotides (nts)/ cycle as estimated by DNA adduct kinetics in HepaRG cells (Yang et al. 2021). It would, therefore, require daily exposure to ES over 6–57 years to yield 10–100 adducts/10^8^ nts (Yang et al. 2021). Noteworthy, a hypolinearity of DNA adduct formation was reported in PRH exposed to low ES concentrations < 1 µM (Schulte-Hubbert et al. 2020), which should thus reduce the calculated rate of DNA adduct accumulation. Whether E3′-*N*^2^-dG adducts also occur in human liver biopsies is currently unknown. However, the presence of hepatic E3′-*N*^2^-dG adducts is very likely, since DNA adducts derived from the structurally related ME were detected in human liver biopsies with a median of 13 adducts/10^8^ nts (Herrmann et al. 2013).

The above-mentioned estimated level of 100 E3′-*N*^2^-dG adducts/10^8^ nts for chronic (lifetime) exposure (i.e., 56 years) is a factor of 2.3–22.3 below the level, which was required to cause clastogenicity in our human liver cell models and thus rather unlikely to occur in humans. In line with this assumption, clastogenicity was not detected in mice exposed to ES (up to 300 mg/kg bw and day) over 13 weeks (Suzuki et al. 2012b). ES was also negative in a study performed according to the OECD test guideline 474, in which rats received up to 2 g ES/kg bw by oral gavage without significant increase in MN levels (Nesslany et al. 2010). More than 60-fold higher adduct levels (~ 6000 E3′-*N*^2^-dG adducts/10^8^ ncs) were necessary to cause cytotoxicity in our human liver cell models, which is very unlikely to happen in humans following chronic dietary exposure to ES. As mentioned above, the low acute toxicity despite high adduct levels is very likely attributable to an efficient bypass of the ES-derived DNA adducts by DNA translesion polymerases, which has been recently demonstrated (Deshmukh et al. 2023).

Importantly, replication across E3′-*N*^2^-dG adducts was catalysed in vitro by human pol κ and pol η in an error-free manner (Deshmukh et al. 2023), i.e., without the misincorporation of wrong nucleotides that could give rise to point mutations. These findings indicate a rather limited mutagenic potential for ES-derived DNA adducts. Consistent with this notion, mutagenicity of ES was observed in male F344 *gpt* delta rats with a significant twofold increase vs. control only at the top dose of 200 mg ES/kg bw for 4 weeks (Suzuki et al. 2012a). This was also confirmed in male *gpt* delta mice, whereas in female *gpt* delta mice, a significant increase in mutation frequency was detected at a lower dose of 75 mg/kg bw (Suzuki et al. 2012b). Interestingly, the higher mutagenicity in female *gpt* delta mice correlated well with their *SULT1A1* expression levels, which were up to fivefold higher than in male *gpt* delta mice (Suzuki et al. 2012b). The relevance of *SULT1A1* expression was also highlighted in a previous study, which displayed a positive association between ME-derived DNA adducts in human liver tissue and the *SULT1A1* copy number (Tremmel et al. 2017). In both *gpt* delta rodent studies mentioned above, hepatic DNA adduct levels were measured as well. The analysis showed clear adduct formation already at an ES dose of 20 and 37.5 mg/kg bw and day, respectively, corresponding to an ES-derived adduct level between 1000 and 3000/10^8^ nts (Suzuki et al. 2012a, 2012b). Notably, at these dose levels, no significant increase in mutation frequency was observed, indicative of a non-linear dose–response relationship for ES-induced mutagenicity.

Non-linear dose–response relationships were already established for alkylating agents, such as *N*-nitroso compounds (NOC), which give rise to DNA alkylation adducts including *O*^6^-methylguanine (*O*^6^-MeG) (Fahrer and Christmann 2023). Non-linearity in NOC-triggered *O*^6^-MeG adduct formation and subsequent carcinogenesis was demonstrated in vivo*,* which was dependent on DNA repair catalysed by *O*^6^-methylguanine DNA methyltransferase (MGMT) (Fahrer et al. 2015; Kraus et al. 2019). Furthermore, non-linearity in mutagenicity and carcinogenicity was reported for heterocyclic aromatic amines (HAA), undergoing metabolic activation via CYP1A2 and SULT1A1 (or NAT2) (Turesky 2007), similar to ES and other phenylpropenes. While the dose–response curve for HAA-dependent DNA adduct formation was linear, non-linearity was observed for mutations, preneoplastic lesions, and tumour formation in rodents, revealing a higher PoD for mutagenicity and carcinogenicity (Fukushima et al. 2004, 2002; Hoshi et al. 2004).

In conclusion, our quantitative genotoxicity study elucidated for the first time that a certain ES-derived DNA adduct level must be present to give rise to clastogenicity and, at even higher levels, cytotoxicity in human liver cells. Despite the reported DNA adduct accumulation, these thresholds of adduct levels are very likely not reached by chronic dietary exposure. Further research is warranted to study the involved DNA repair pathways and the occurrence of E3′-*N*^2^-dG adducts in human liver tissue.

## Supplementary Information

Below is the link to the electronic supplementary material.

Supplementary file1 (DOCX 3533 KB)

## Data Availability

All datasets generated and analysed during this study were included in the manuscript and the supplementary information. They are also available from the corresponding author upon reasonable request.
