# Supplementary material for: Molecular dosimetry of estragole and 1′-hydroxyestragole-induced DNA adduct formation, clastogenicity and cytotoxicity in human liver cell models
Source: Arch Toxicol. 2025 May 21;99(9):3769–85. doi: 10.1007/s00204-025-04084-2 (PMC12408683; doi:10.1007/s00204-025-04084-2)
Supplement: Supplementary file 1 — Supplementary file1 (DOCX 3533 KB) [file 204_2025_4084_MOESM1_ESM.docx]

**Supplementary Figures**

**Figure 1**

**Fig. 1: Characterization of the synthesized 1′OH-ES via ^1^H-NMR and ^1^H-^1^H-COSY-NMR. A**: ^1^H NMR (400 MHz, DMSO-*d*_6_) δ 7.27 – 7.17 (m, 2H), 6.91 – 6.83 (m, 2H), 5.92 (ddd, *J* = 17.1, 10.2, 5.8 Hz, 1H), 5.36 (d, *J* = 4.4 Hz, 1H), 5.20 (ddd, *J* = 17.1, 2.1, 1.5 Hz, 1H), 5.05 – 4.96 (m, 2H), 3.73 (s, 3H). **B**: ^1^H-^1^H-COSY-NMR of 1′OH-ES.

**Figure 2**

**Fig. 2: UHPLC-MS/MS method for the detection of estragole-derived DNA adducts. A:** Gradient of the UHPLC method to separate the two main DNA adducts E3′-*N*^6^-dA and E3′-*N*^2^-dG. **B:** Gradient of the HPLC method to detect dG.

**Figure 3**

**
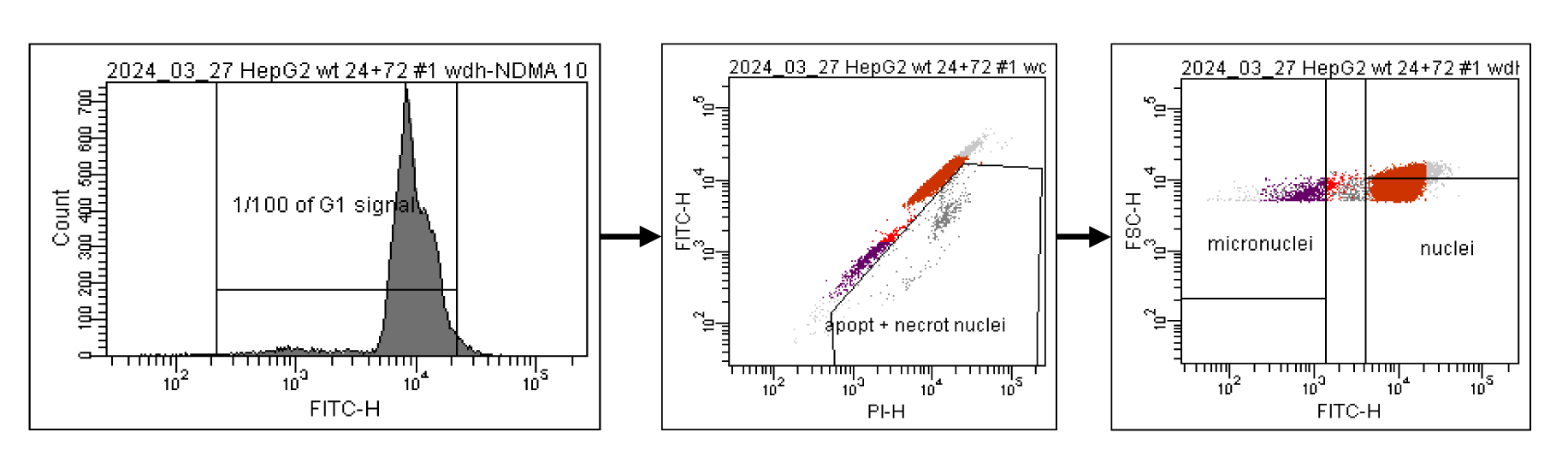
**

**Fig. 3: Gating strategy for the flow cytometry-based micronucleus assay.** Initially, signals with a SYTOX Green signal below 1/100 of the G1-Phase are discriminated. Next, EMA positive nuclei are discriminated to exclude dead cells. Finally, intact G1-phase cells and micronuclei are depicted. Signal from micronuclei is not greater than 1/3 of the G1-phase nuclei signal and, thus, the distance between the two gates (MN and nuclei) was adjusted accordingly.

**Figure 4**

**
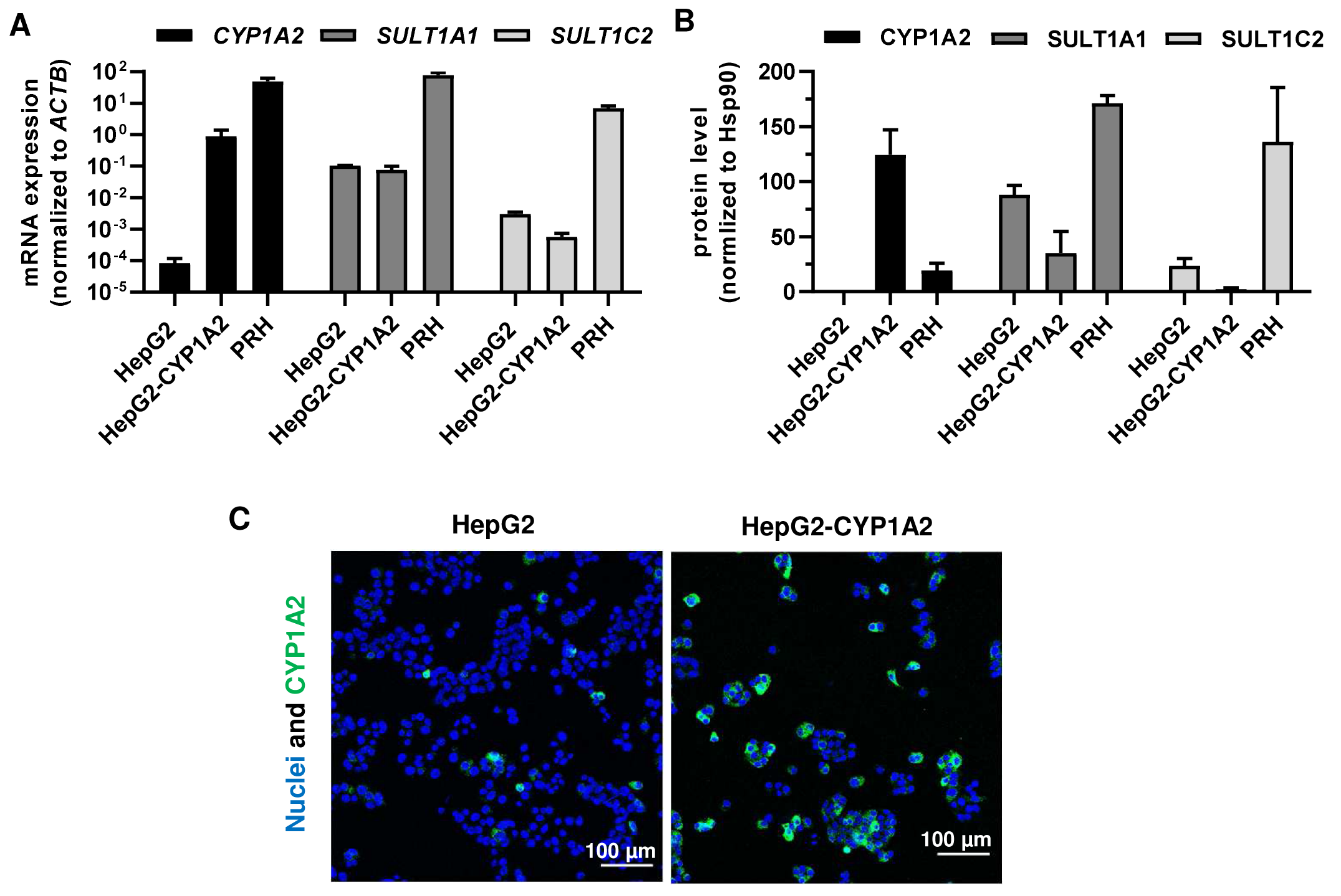
**

**Fig. 4: Metabolic competence of the used cell models. A** Gene expression levels of *CYP1A2*, *SULT1A1* and *SULT1C2* in HepG2, HepG2-CYP1A2 and primary rat hepatocytes (PRH) determined by qPCR (n=3). **B:** Protein expression levels of CYP1A2, SULT1A1 and SULT1C2 in HepG2, HepG2-CYP1A2 and PRH assessed by western blot analysis (n=3). **C:** Confocal microscopy of CYP1A2 in HepG2 and HepG2-CYP1A2. Representative images are shown.

**Figure 5**

**
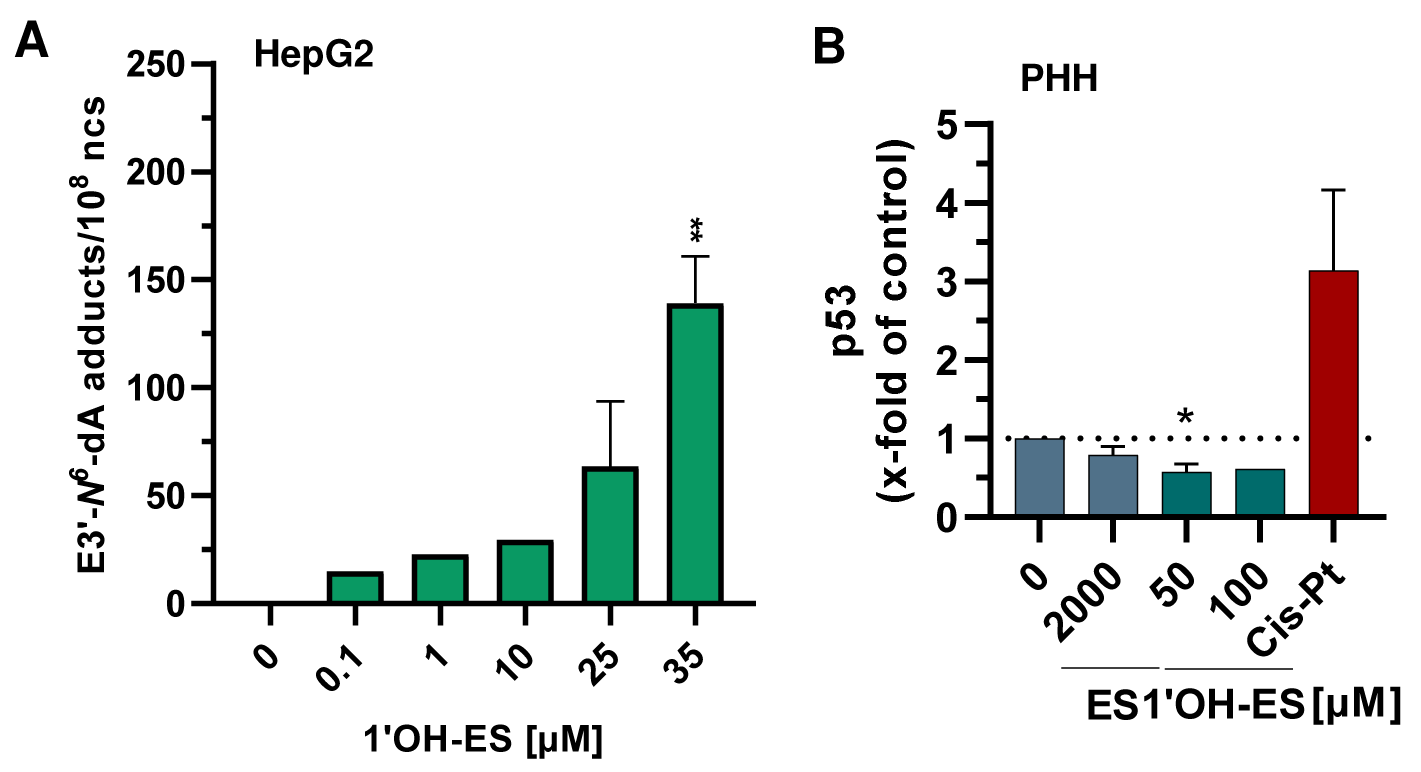
**

**Fig. 5: E3′-*N*^6^-dA formation in HepG2 cells after 1′OH-ES treatment and p53 level in primary human hepatocytes.** **A** Quantification of E3′-*N*^6^-dA adducts in HepG2 cells after treatment with 1′OH-ES for 24 h (n=4). The data are given as mean + SEM, except for concentrations ≤ 10 µM with some samples below the LOQ and, thus, only the mean is shown. Statistical analysis was performed using two tailed, unpaired t-test versus the theoretical LOQ (**p < 0.01). **B** Western blot analysis of p53 levels in primary human hepatocytes (PHH) exposed to estragole (ES) and 1′OH-ES as indicated for 24 h (n=3, except for 100 µM with n=2). DMSO was used as solvent control, whereas cisplatin (Cis‑Pt, 10 µM) served as positive control. p53 levels were determined in relation to Hsp90 as loading control and are presented as mean + SEM. Statistical analysis was performed using two tailed, unpaired t-test versus the solvent control (*p < 0.05).

**Figure 6**

**
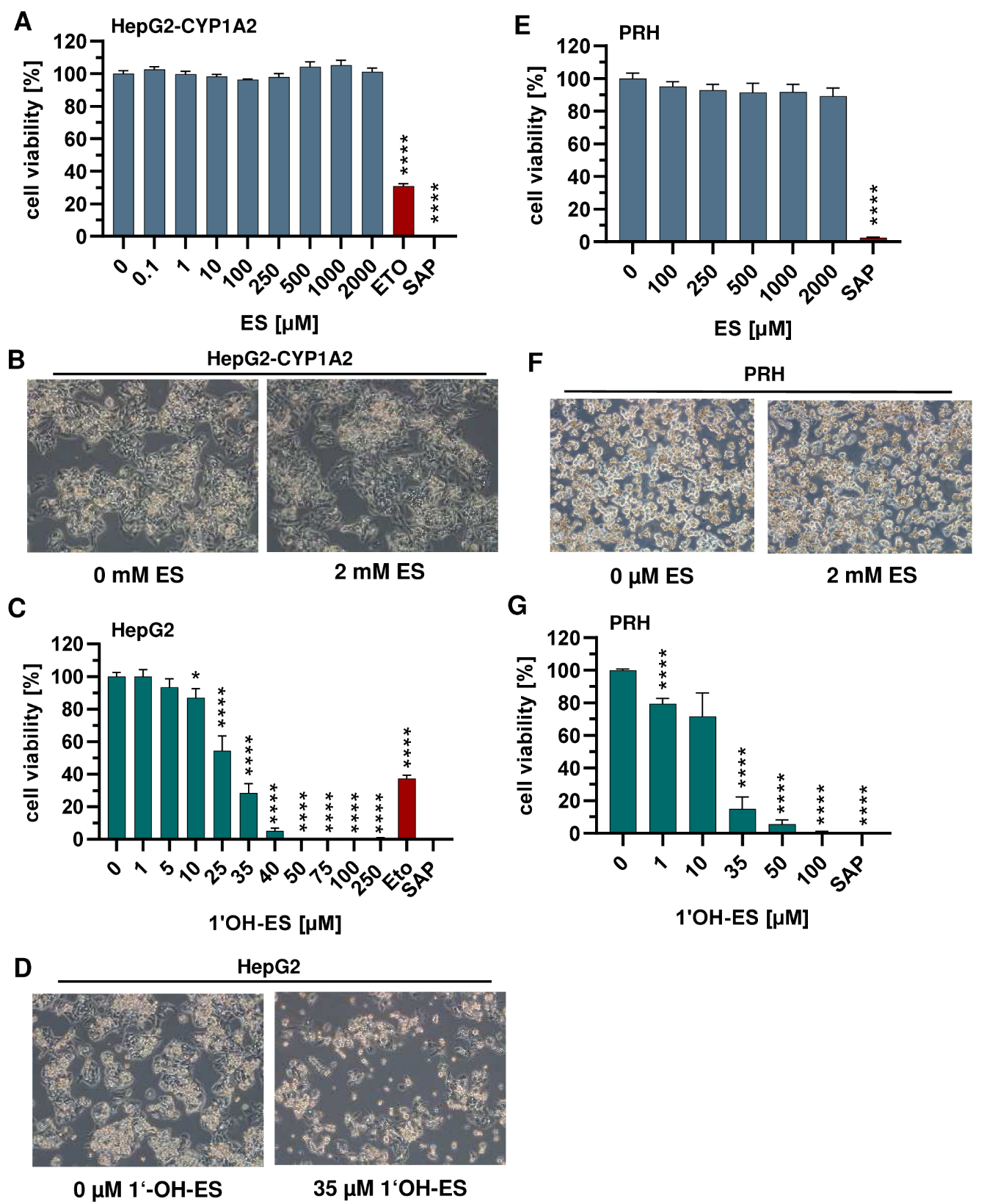
**

**Fig. 6: Cytotoxic effects of ES and 1′OH-ES in HepG2 liver cell models and primary rat hepatocytes. A** Viability of HepG2-CYP1A2 cells after treatment with estragole (0–2 mM) for 72 h (n=3). **B** Representative images of treated HepG2-CYP1A2 cells (10x magnification). **C** Viability of HepG2 cells after treatment with 1′OH-ES (0–250 µM) for 72 h (n≥3). **D** Representative images of treated HepG2 cells (10x magnification). **E** Viability of primary rat hepatocytes (PRH) after treatment with estragole (0–2 mM) for 24 h (n=3). G Representative images of treated PRH (10x magnification). **G** Viability of PRH after treatment with 1′OH-ES (0-250 µM) for 24 h (n=3). Saponin (SAP, 0.1 %) and etoposide (ETO, 10 µM) were used as positive controls, whereas DMSO served as solvent control. All data given as mean + SEM. Statistical analysis was performed using two tailed, unpaired t-test versus the solvent control (*p < 0.05, ****p < 0.0001).

**Figure 7**

**
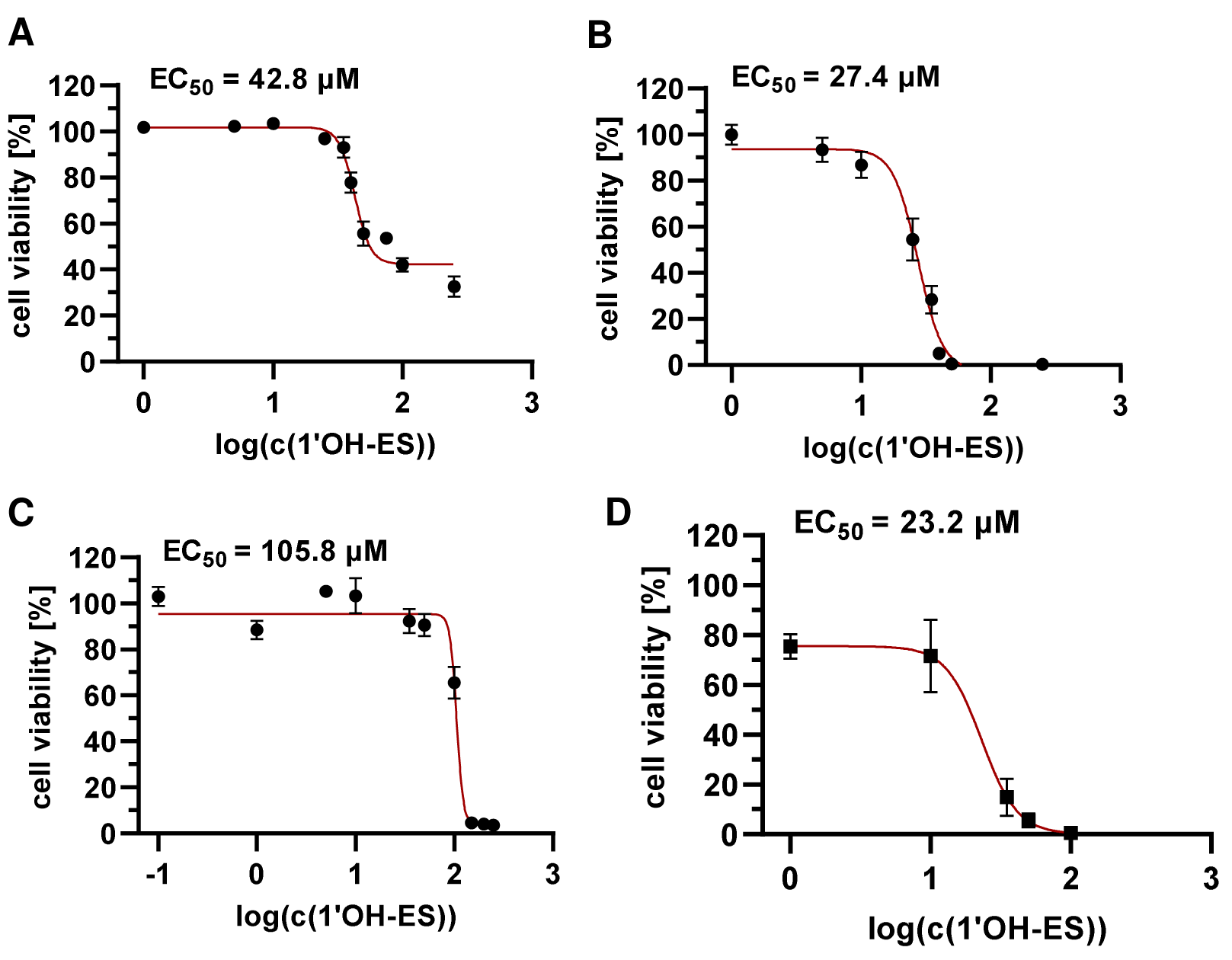
**

**Fig. 7: Non-linear regression to calculate EC_50_ values for 1′OH-ES in different liver cell models.**

**A-D:** Determination of EC_50_ values based on cell viability data in HepG2 cells (A and B), PHH and PRH after exposure to 1′OH-ES over 24 (A, C and D) and 72 h (B). Concentrations were transformed into the log scale and plotted against viability. The data were then fitted by nonlinear regression with variable slope using GraphPad Prism 8.

**Figure 8**

**
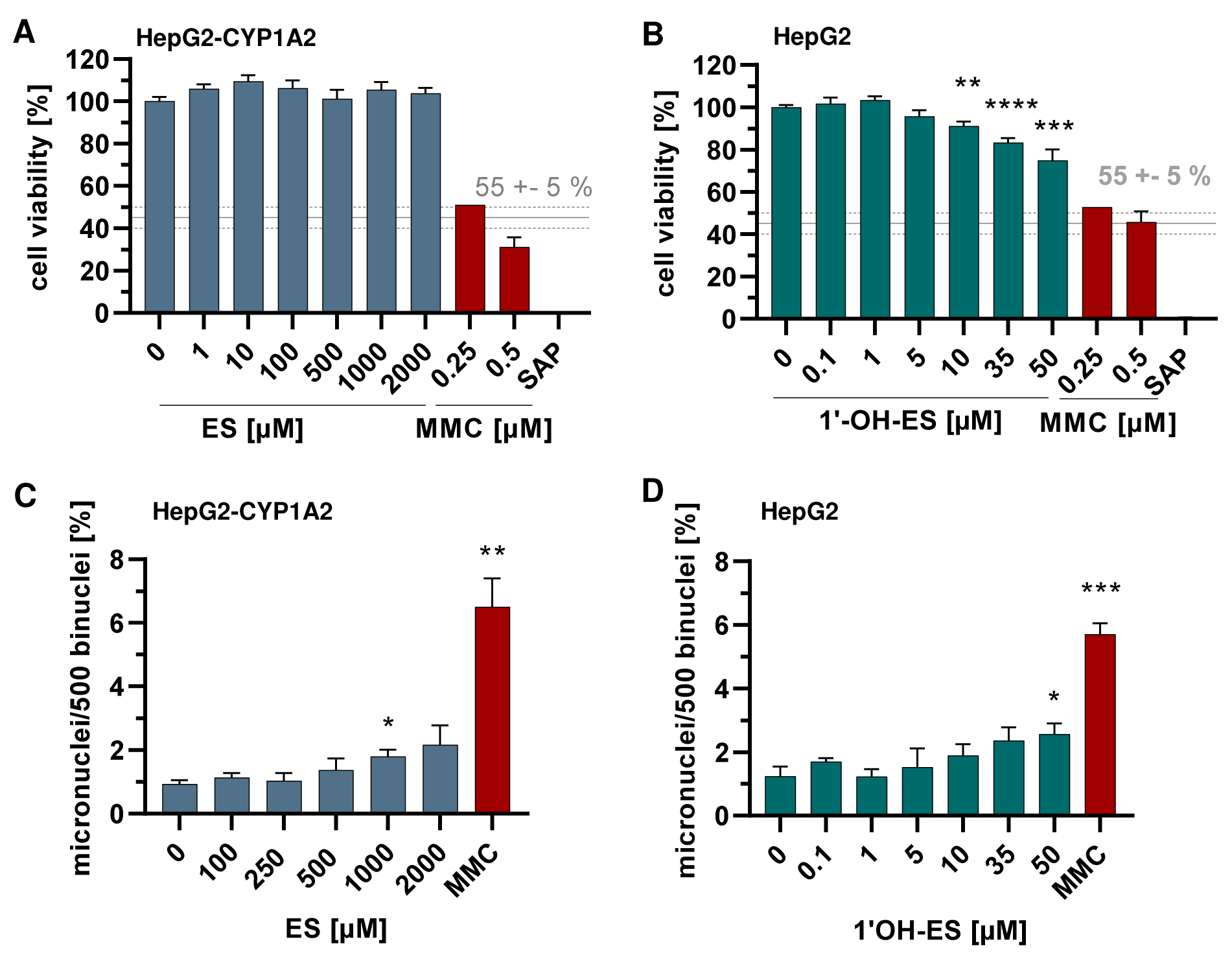
**

**Fig. 8: Cell viability and cytokinesis block micronucleus assay in HepG2 liver cell models after exposure to ES and 1**′**OH-ES.** **A and B** Cytotoxicity after treatment with ES (A) and 1′OH-ES (B) for 24 h and 72 h recovery time in HepG2-CYP1A2 cells (A) and HepG2 cells (B). Mitomycin C (MMC) and saponin (SAP; 0.1 %) were used as positive controls (n=3, except for 0.25 µM MMC with n=2). **C** and **D** Cytokinesis block micronucleus assay after treatment with ES (C) and 1′OH-ES (D) for 24 h and 72 h recovery time in HepG2-CYP1A2 cells (C) and HepG2 cells (D). MMC and SAP (0.1 %) were used as positive controls (n≥3). All data shown as mean + SEM. Statistical analysis was performed using two tailed, unpaired t-test versus solvent control (*p < 0.05, **p < 0.01, ***p < 0.001, ****p < 0.0001).

**Figure 9**

**
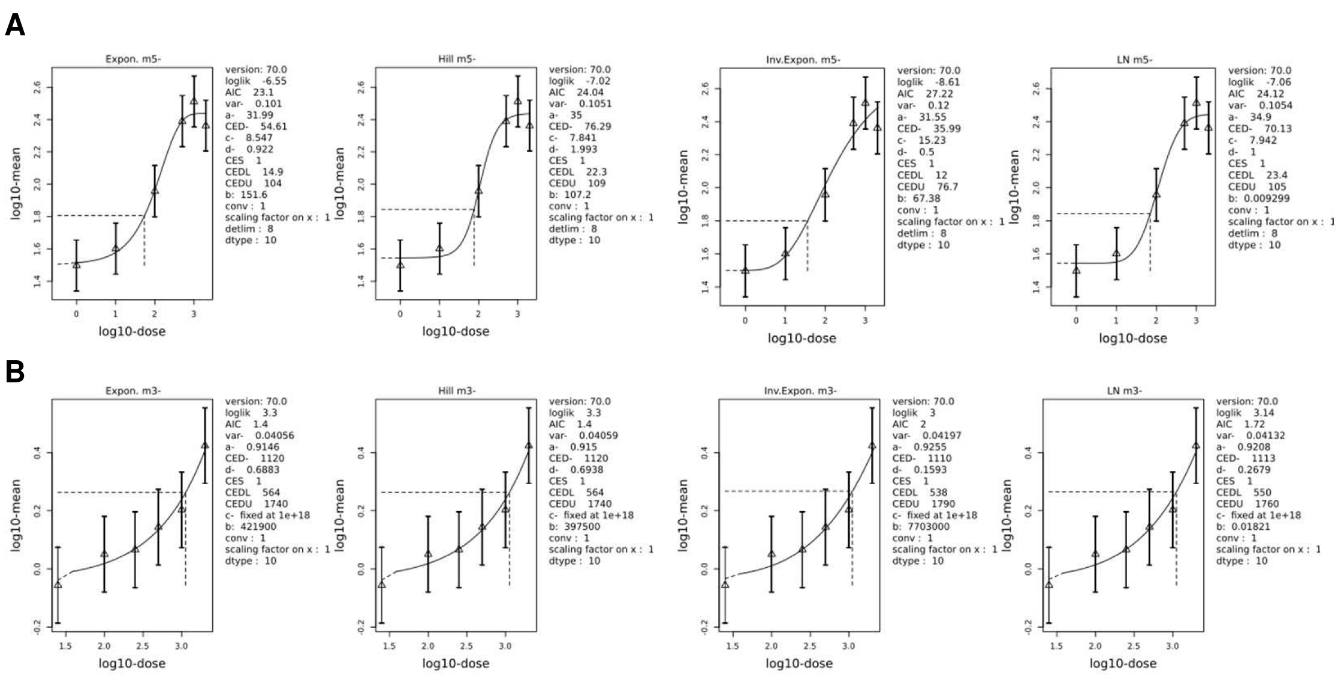
**

**Fig. 9: BMC modeling of concentration-response data for ES. A** E3′-*N*^2^-dG adduct data. **B** Micronuclei data (CBMN assay). Shown are the four best fits used for model averaging.

**Figure 10**

**
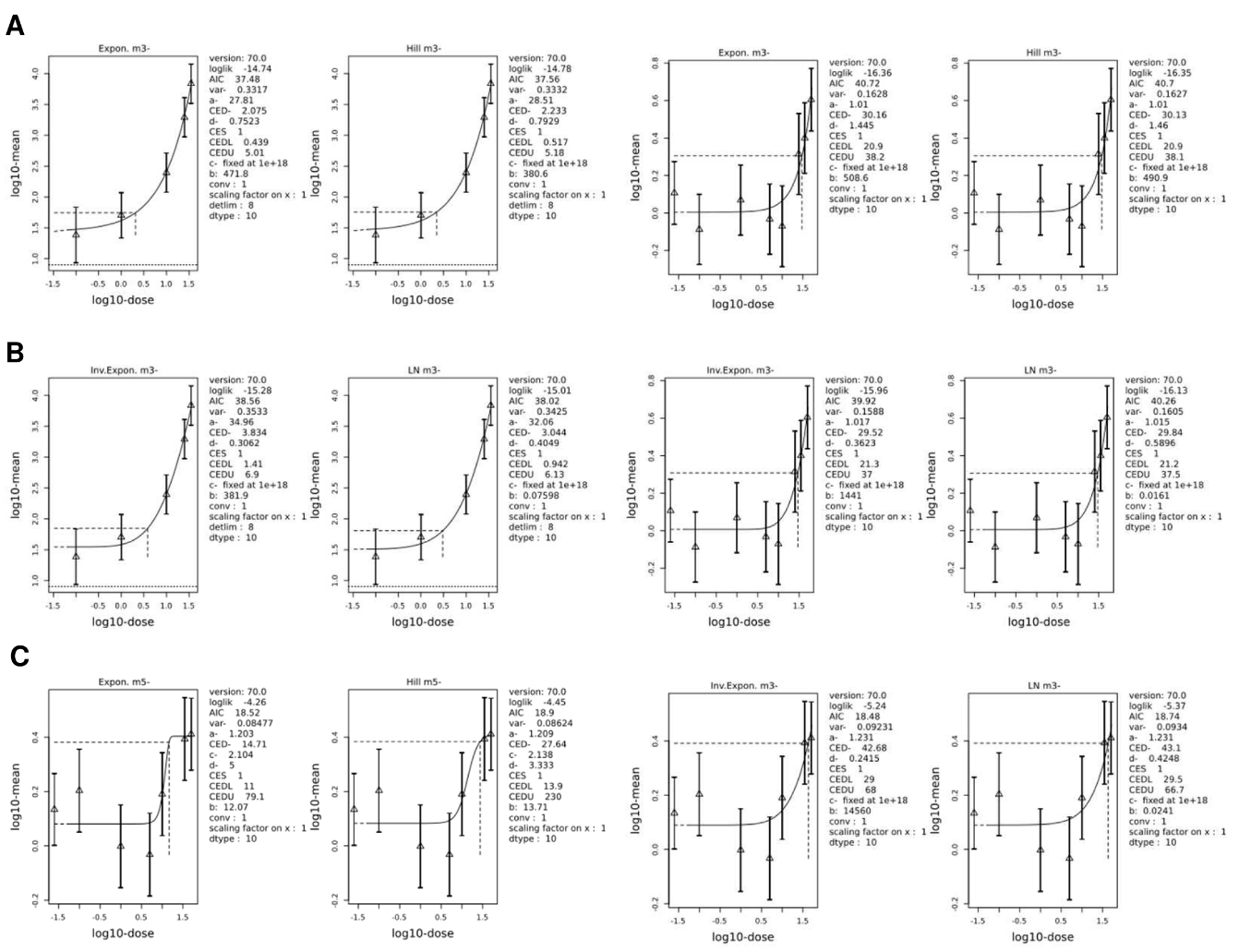
**

**Fig. 10: BMC modeling of concentration-response data for 1**′**-OH-ES. A** E3′-*N^2^*-dG adduct data. **B** Micronuclei data (flow cytometry) **C** Micronuclei data (CBMN assay). Shown are the four best fits used for model averaging.

**Supplementary Tables**

**Table S1: LOD and LOQ of the UHPLC-MS/MS method to quantify E3**′**-*N^2^*-dG and E3**′**‑*N^6^*‑dA.**

|  | **E3**′**-*N^2^*-dG** | **E3**′**-*N^6^*-dA** |
| --- | --- | --- |
| **LOD**  (3x Signal-to-Noise) | 74.5 pM  (≈ 4 adducts/10^8^ nucleosides) | 32.8 pM  (≈ 2 adducts/10^8^ nucleosides) |
| **LOQ**  (6x Signal-to-Noise) | 149 pM  (≈ 8 adducts/10^8^ nucleosides) | 78.7 pM  (≈ 4 adducts/10^8^ nucleosides) |

**Table S2: Compound specific MS parameters.** Q1, quadrupole 1; Q3, quadrupole 3; DP, declustering potential; EP, entrance potential; CEP, collision cell entrance potential; CE, collision energy; CXP, cell exit potential.

|  | **Q1**  **[*m/z*]** | **Q3**  **[*m/z*]** | **DP**  **[V]** | **EP**  **[V]** | **CEP**  **[V]** | **CE**  **[V]** | **CXP**  **[V]** |
| --- | --- | --- | --- | --- | --- | --- | --- |
| dG1 | 268.2 | 151.9 | 6 | 4.5 | 12 | 15 | 18 |
| dG2 | 268.2 | 134.9 | 6 | 4.5 | 12 | 47 | 20 |
| ^15^*N*_5_-dG1 | 273.1 | 157.3 | 26 | 4 | 20 | 17 | 4 |
| ^15^*N*_5_-dG2 | 273.1 | 138.9 | 26 | 4 | 20 | 49 | 4 |
| E3‘-*N*^2^-dG1 | 414.0 | 298.0 | 36 | 10 | 20 | 15 | 24 |
| E3‘-*N*^2^-dG2 | 414.0 | 163.8 | 36 | 10 | 20 | 31 | 18 |
| ^15^N_5_-E3‘-*N*^2^-dG1 | 419.0 | 303.1 | 46 | 10 | 20 | 13 | 14 |
| ^15^N_5_-E3‘-*N*^2^-dG2 | 419.0 | 168.9 | 46 | 10 | 20 | 33 | 12 |
| E3‘-*N*^6^-dA1 | 398.3 | 147.1 | 91 | 10 | 20 | 33 | 10 |
| E3‘-*N*^6^-dA2 | 398.3 | 281.9 | 91 | 10 | 20 | 19 | 32 |
| ^15^N_5_-E3‘-*N*^6^-dA1 | 403.2 | 147.1 | 41 | 10 | 20 | 39 | 14 |
| ^15^N_5_-E3‘-*N*^6^-dA2 | 403.2 | 287.2 | 41 | 10 | 20 | 19 | 14 |

**Table S3: Antibodies used in this study**

| **Antibody** | **Catalog No.** | **Provider** |
| --- | --- | --- |
| Anti-p53 (DO1), mouse monoclonal | sc-126 | Santa Cruz Biotechnology,  Heidelberg, Germany |
| Anti-γH2AX, rabbit monoclonal | ab81299 | Abcam, Cambridge, UK |
| Anti-Hsp90α/β (F-8), mouse monoclonal | sc-13119 | Santa Cruz Biotechnology,  Heidelberg, Germany |
| Anti-CYP1A2 (D15), mouse monoclonal | sc-53241 | Santa Cruz Biotechnology,  Heidelberg, Germany |
| Anti-SULT1C2, rabbit polyclonal | ab243329 | Abcam, Cambridge, UK |
| Anti-SULT1A1, polyclonal rabbit | GTX55811 | Genetex, Irvine, California, USA |
| mouse IgGκ binding protein-HRP | sc-516102 | Santa Cruz Biotechnology,  Heidelberg, Germany |
| Goat-anti-Rabbit-HRP | #7074 | Cell Signaling Technology,  Danvers, Massachusetts, USA |
| F(ab’)2-Goat-anti-Mouse IgG (H-L) Alexa Fluor 488 | A-11017 | Life Technologies, Darmstadt, Germany |

**Table S4: Used primer sequences to determine *CYP1A2*, *SULT1A1* and *SULT1C2* expression levels by qPCR**

| **qPCR Primer** | **Sequence (5‘-3‘)** |
| --- | --- |
| hCYP1A2-for | TTCGTAAACCAGTGGCAGGT |
| hCYP1A2-rev | AGGGCTTGTTAATGGCAGTG |
| hSULT1A1-for | CAAAGGATGTGGCAGTTTCC |
| hSULT1A1-rev | CCGACCATGAACTTCTCCAG |
| hSULT1C2-for | GGAGCCAGATCCAGAGCTTC |
| hSULT1C2-rev | CGTCCCCATTCTGTTCAATC |
| hACTB-for | tggcatccacgaaactacc |
| hACTB-rev | gtgttggcgtacaggtctt |
| hGAPDH-for | catgagaagtatgacaacag |
| hGAPDH-rev | atgagtccttccacgata |
| rCYP1A2-for | CCTTGGGCTCTGTCACAAGT |
| rCYP1A2-rev | GGTCTCATCCCTCAGGAGAA |
| rSULT1A1-for | CAGCTTGGCCATGTTGTAGA |
| rSULT1A1-rev | GTCCTTGCTCCCTCAGAGTC |
| rSULT1C2-for | CTACATCCCCATTCTGCTCAA |
| rSULT1C2-rev | TCAGACCTTCAAGGCGAAG |

**Table S5: Weighting of BMC modeling**

|  | **Endpoint** | **Exp** | **Hill** | **Invexp** | **logn** |
| --- | --- | --- | --- | --- | --- |
| **ES** | E3′-*N^2^*-dG | 0.425 | 0.2656 | 0.0542 | 0.2552 |
|  | MN | 0.2783 | 0.2783 | 0.2062 | 0.2372 |
| **1**′**OH-ES** | E3′-*N^2^*-dG | 0.3024 | 0.2905 | 0.1762 | 0.2308 |
|  | MN (FCM) | 0.2101 | 0.2122 | 0.3134 | 0.2644 |
|  | MN | 0.2672 | 0.2209 | 0.2726 | 0.2393 |
